# A Lightweight Hybrid-Attention Conformer for Affective State Recognition from Wearable EEG

**DOI:** 10.3390/s26216943

**Published:** 2026-11-03

**Authors:** Mustafa Eriş, Fatma Güneş Eriş, Orhan Yaman

**Affiliations:** Department of Digital Forensics Engineering, Faculty of Technology, Fırat University, Elazığ 23119, Türkiye; meris@firat.edu.tr (M.E.); orhanyaman@firat.edu.tr (O.Y.)

**Keywords:** affective computing, emotion recognition, electroencephalography (EEG), raw EEG classification, conformer, hybrid attention, wearable EEG sensor, consumer-grade EEG, edge computing, deep learning

## Abstract

Recognizing affective states from electroencephalography (EEG) is a core task in affective computing, with applications in mental health monitoring and public safety. Most existing approaches rely on handcrafted features or image-based transformations of EEG, while transformer models capture global context at high computational cost. This study proposes KimiFormer, an end-to-end hybrid-attention Conformer that classifies raw EEG directly. A hierarchical Spatio-Temporal Stemming (STS) module tokenizes the multichannel signal and learns inter-electrode relationships. Stacked Conformer blocks combine linear and full attention in a 3:1 ratio, reducing computational cost while preserving long-range modeling. The model was evaluated on the public EEG Violence Dataset, recorded with a 14-channel wearable headset from 14 participants viewing violent and neutral videos. Under record-wise leave-one-record-out cross-validation with nested selection of the training hyperparameters, KimiFormer achieved 95.5% accuracy (97.0% under 10-fold cross-validation). On the external DREAMER dataset, its subject-independent performance remained close to chance (balanced accuracy 52.7% for valence and 50.2% for arousal), as did that of two established raw-EEG models. Ablation showed the STS module to be essential (65% accuracy without it). With 3.2 M parameters, 1.51 GFLOPs and an inference latency of 15.64 ms per sample, KimiFormer is suited to real-time, on-device processing of low-channel wearable EEG.

## 1. Introduction

### 1.1. Background

The rapid advancement of brain–computer interface (BCI) technology has made it possible to more successfully transfer signals obtained from the human brain into a digital environment [1]. Electroencephalography (EEG) signals are non-invasive electrical recordings of brain activity that capture neural oscillations and temporal dynamics. By recording brain activity over time, EEG signals provide valuable information for understanding cognitive states, emotional responses, and motor intentions [2]. EEG signals can be used as an important resource for understanding changes in an individual’s emotional state or dominant emotions [3]. Affective computing systems increasingly combine brain, physiological and behavioral signals. Among these, EEG provides the most direct measure of the neural processes that produce emotional responses, so accurate and efficient EEG models are a key building block for multimodal emotion recognition. Consumer-grade wearable EEG headsets make continuous affective monitoring outside the laboratory feasible, but their few channels, saline or dry electrodes, low sampling rates and susceptibility to motion artifacts give a much lower signal quality than clinical systems. Models for such sensors must therefore learn directly from noisy raw signals while remaining light enough for on-device inference.

**Figure 1:**
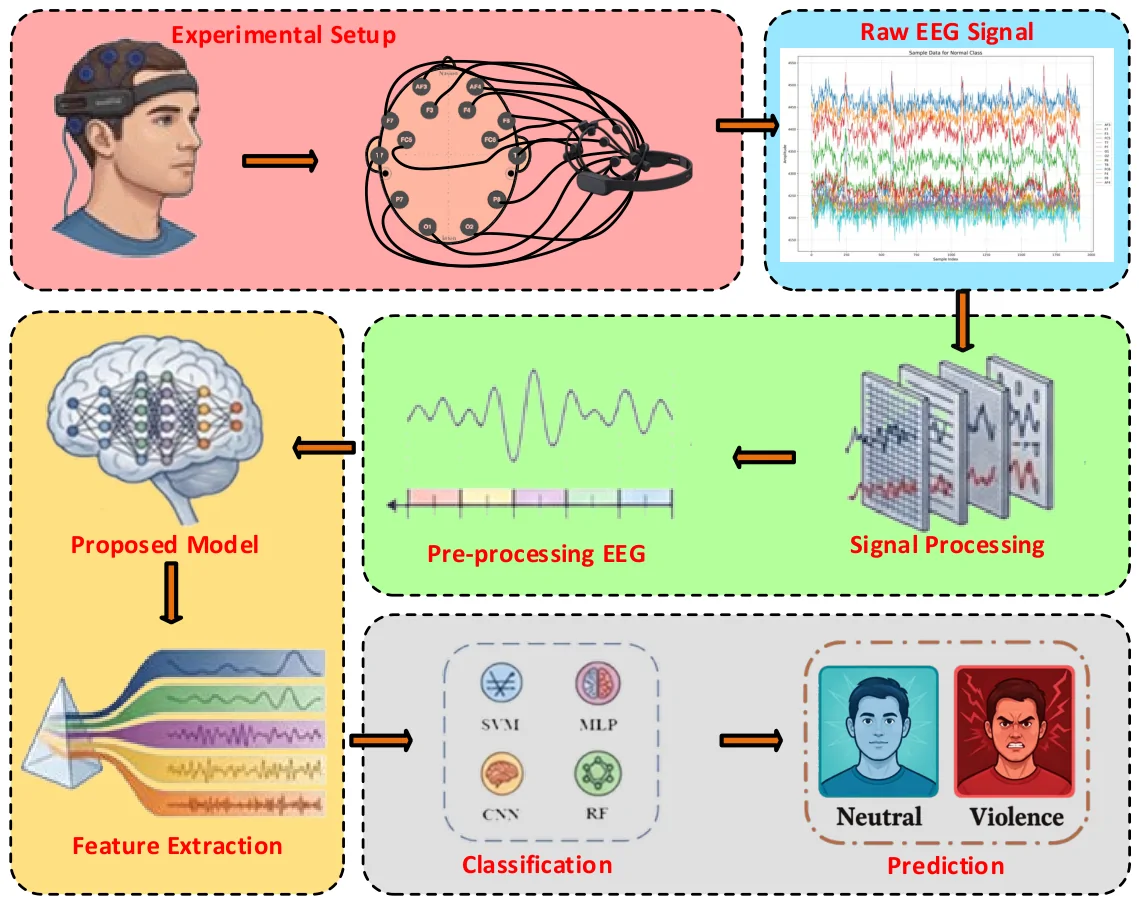
The overall workflow of EEG-based signal processing systems.

Stress and aggressive behavior are critical public health and safety issues [4]. If these situations are ignored at an early stage, chronic stress can lead to serious health problems. Uncontrolled aggression also threatens public safety in areas such as workplaces and schools. Therefore, developing automated systems to detect these states early is essential for preventing such consequences [5]. In recent years, EEG-based studies for stress and aggression detection have gained more attention to address these challenges. Researchers have explored various signal processing and machine learning techniques to identify patterns related to stress and aggressive behavior. These studies aim to provide objective, real-time assessment of mental states, reducing reliance on subjective self-reports. As a result, EEG-based automated detection systems show strong potential for supporting early intervention and improving mental health monitoring [6,7,8,9]. The typical pipeline for automatic EEG signal processing has several sequential stages such as signal preprocessing, feature extraction, model training, and prediction. The general structure of EEG signal studies is given in Figure 1.

However, analyzing raw EEG signals is a challenging task [10]. EEG data is complex, contains significant noise (low signal-to-noise ratio), and varies greatly between different individuals. Traditional machine learning methods struggle to process this complex data because they rely on manual feature extraction, which is time-consuming and may be dependent on the specific task.

In recent years, deep learning models have improved EEG analysis. However, deep learning models also have limitations. Convolutional neural networks (CNNs) are good at detecting local patterns but often miss long-term temporal dependencies. Recurrent neural networks (RNNs) can handle time sequences but become inefficient when the input signal is long. Although transformers have improved time-series analysis, applying a standard vision transformer (ViT) to raw EEG is computationally expensive. Furthermore, transformers often fail to capture local signal details effectively. To overcome these challenges, we propose KimiFormer, a novel hybrid architecture designed for raw EEG classification. KimiFormer combines the efficiency of convolutions with the power of transformers to effectively process raw EEG signals.

Literature Gap: 

In the literature, it is clear that hybrid models achieve better performance than single models. However, a common limitation across many studies is the use of complex feature engineering processes or reliance on converting EEG signals into images. Developing end-to-end hybrid models that can capture temporal and spatial features directly from raw EEG signals is considered an open research area. In addition, optimizing the computational cost of these models is an important direction for future research. This study aims to address this gap by enabling direct learning from raw signals using a newly proposed hybrid attention mechanism.

Main Contributions:

Existing research frequently struggles with the trade-off between computational efficiency and feature extraction capability, even with the advances in deep learning-based EEG analysis techniques. Furthermore, manual feature transformations like time-domain statistics, frequency-domain representations, or time-frequency decompositions are used in the majority of the studies. The model’s capacity to directly learn discriminative features from unprocessed EEG signals may be constrained by this circumstance. The primary contributions of this work are summarized as follows:

Novelties:

- We introduced a novel hierarchical tokenization module, Spatio-Temporal Stemming (STS), to process and transform raw EEG data into rich context embeddings.
- To hierarchically capture local spatiotemporal correlations, the STS block uses convolutions. Additionally, this block directly extracts robust features from raw EEG signals.
- We proposed a hybrid attention Conformer block to balance the computation cost and feature extraction ability of the proposed model.
- The suggested KimiFormer model switches between Full Attention and Linear Attention. The quadratic complexity of conventional transformer architectures is lessened by this design.

Contributions:

- We proposed an end-to-end framework that eliminates the need for manual preprocessing or handcrafted feature engineering of raw EEG signals. This simplifies the deployment pipeline for real-time BCI applications.
- Ablation studies indicate that the proposed STS module contributes substantially to EEG classification: removing this module leads to a substantial decrease in performance. This highlights the importance of local feature extraction in Transformer-based EEG models.
- We evaluate the model with segment-wise 10-fold and record-wise leave-one-record-out (LORO) cross-validation (97.0% and 95.5% accuracy, the latter with nested selection of the training hyperparameters) and evaluate it on the external DREAMER dataset under a leave-one-subject-out (LOSO) protocol.

### 1.2. Literature Review

EEG signals, which reflect the electrical activity of the human brain, are considered one of the most reliable biomarkers in areas such as stress detection, emotion recognition, and determining violent tendencies. Various studies have been conducted in the literature on the detection and classification of stress and violence using EEG signals. A review of the literature reveals that studies in this field have evolved from traditional feature engineering to complex deep learning architectures that enable end-to-end learning.

Early and some recent studies have focused on extracting statistical, temporal, or frequency-based features from EEG signals, followed by the use of traditional classifiers. For example, Chhabra et al. performed signal decomposition using the wavelet transform, extracted entropy-based features, and achieved a classification accuracy of 94% using a support vector machine (SVM) classifier [11]. Similarly, Fernandez et al. proposed a computationally efficient and high-performing model by classifying simple statistical features of EEG signals, such as the mean and standard deviation, using the LightGBM algorithm as an alternative to the complexity of deep learning models [12].

In recent years, explainable artificial intelligence and feature engineering-based models have gained attention to improve the interpretability of model decisions. Bektas et al. proposed the “Channel-based Min–Max Pattern” (ChMinMaxPat) method for violence and stress detection. They achieved over 99% accuracy in violence detection, but the performance for stress detection remained at 73.30% under LORO validation [13]. Using a similar approach, İnce et al. developed the “Cubic Pattern” (CubicPat) method, which encodes EEG channels in three dimensions for mental performance and stress analysis, and achieved an accuracy of 96.29% using 10-fold cross-validation [14]. Cambay et al. used the “Quadruple Transition Pattern” (QuadTPat) method and explained the role of the frontal and temporal lobes in stress responses using the DLob symbolic language [15].

To improve efficiency in wearable devices, Shikha et al. proposed a framework called KRAFS-ANet. In this study, channel selection was performed using the normalized mutual information (NMI) and recursive feature elimination (RFE) algorithms, followed by a stacking architecture consisting of Bagging (k-nearest neighbors (KNN), random forest (RF) and SVM) and an artificial neural network (ANN) meta-classifier, achieving an accuracy of 98.63% on the MAT dataset [16].

Deep learning models are becoming standard in EEG analysis due to their ability to automate feature extraction. In particular, CNNs are widely used to capture the spatial characteristics of EEG data. Afify et al. adapted the VGGish model, originally developed for audio classification, to EEG data using transfer learning and achieved an accuracy of 99.25% in classifying stress tasks on the SAM 40 dataset [17]. Bhatnagar et al. used EEGNet, a compact CNN architecture, to determine stress levels in adolescents under music intervention and reported an accuracy of 99.45% [18].

In stress detection, approaches that process data by transforming it into different formats have also gained attention. Mane and Shinde, in their model called “StressNet,” converted EEG signals into azimuth projection images and achieved an accuracy of 97.8% using a hybrid 2D-CNN and long short-term memory (LSTM) architecture [19]. Similarly, Hafeez and Shakil converted EEG frequency bands into “brainwave images” and classified them using a CNN, achieving a significantly higher accuracy (90.64%) compared to an LSTM model (70.67%) [20].

To overcome the limitations of single models, hybrid architectures have been developed. Modi et al. proposed a hybrid model that integrates CNN and multilayer perceptron (MLP) architectures and reported that it outperformed traditional LSTM and Transformer models [21]. Gonzalez-Vazquez et al. used a gated recurrent unit (GRU)-based RNN model to predict stress levels from EEG data collected during serious games, but reported that the model’s generalizability at the group level remained around 73% [22].

The integration of attention mechanisms improves model performance by enabling the model to focus on the most relevant time segments or channels of the signal. Mukherjee and Roy proposed an attention-based CNN-TLSTM model that uses frontal lobe EEG signals and pulse data. This model classified four different stress levels with an accuracy of 99.72%, demonstrating a clear advantage over traditional machine learning methods such as SVM and KNN [23]. Additionally, Rai and Singh proposed the Attention-based AOS-GAN model and achieved 94% accuracy on noisy EEG data by combining attention-based segmentation (FA-FCN) with adversarial learning [24]. In another study, Arya and Sharma proposed an Internet of Things (IoT)-based system for women’s safety that processed EEG and eye-blink signals using a hybrid RNN/CNN architecture. They achieved an accuracy of 96.8% in threat classification [25].

Beyond studies that target stress or violence specifically, the broader methodological literature on deep learning for EEG is directly relevant to this work. Systematic reviews show that convolutional, recurrent and attention-based networks now dominate EEG decoding, but also that many studies rely on small cohorts, heterogeneous evaluation protocols and within-subject validation, which makes reported accuracies difficult to compare [26,27]; the same observations have been made for EEG-based emotion recognition in particular [28]. End-to-end models that learn directly from raw multichannel EEG were popularized by the shallow and deep ConvNets of Schirrmeister et al. [29] and by EEGNet, a compact network with depthwise and separable convolutions that performs well across paradigms with very few parameters [30]. For affective computing, TSception combines multi-scale temporal kernels with hemisphere-aware spatial kernels to recognize emotions from raw EEG [31].

Transformer-based models have been adapted to EEG to capture long-range temporal dependencies. BENDR uses a transformer with contrastive self-supervised pre-training on large unlabeled EEG corpora [32], and Xie et al. combined convolutional feature extraction with transformer encoders for raw EEG classification [33]. The EEG Conformer of Song et al. couples a convolutional patch-embedding module with multi-head self-attention and has become a common baseline for raw-EEG decoding [34]. A recent review of transformer architectures for motor imagery, seizure and emotion classification identifies hybrid convolution–attention designs as a frequent choice and highlights the limited size of EEG datasets and the computational cost of attention as recurring challenges [35]. KimiFormer belongs to this hybrid family; it differs from EEG Conformer mainly in its hierarchical spatio-temporal stem and in replacing most full-attention layers with linear attention.

Generalization to unseen individuals is the central difficulty for practical EEG systems, because the statistics of the signal differ strongly between subjects and sessions. Transfer-learning approaches for subject-independent brain–computer interfaces have been reviewed by Lotte et al. [36], and a recent systematic review by Apicella et al. organizes cross-subject and cross-session strategies for emotion recognition, such as data alignment, domain adaptation and domain generalization, and stresses that results obtained under LOSO protocols are not comparable with within-subject results [37]. Domain-adaptation methods such as plug-and-play domain adaptation narrow this gap but require data from the target subject for adaptation [38].

Consumer-grade wearable headsets such as the Emotiv EPOC used in this study have been validated against research-grade systems for event-related potentials [39], and a scoping review of research with consumer-grade EEG concludes that these devices can provide research-quality data in multi-trial designs but have a lower signal-to-noise ratio and require careful handling of artifacts [40]. Public affective datasets recorded with such devices, most notably DREAMER, which uses the same 14-channel Emotiv headset and in which all 23 participants experience every stimulus condition [41], and the laboratory-grade DEAP database [42] are the established benchmarks for subject-independent evaluation.

Finally, several studies have shown that the evaluation protocol can dominate the reported performance. Brookshire et al. demonstrated that splitting EEG segments rather than subjects between the training and test sets inflates the accuracy of deep learning models [43], and Kapoor and Narayanan identified such data leakage as a widespread cause of irreproducible results in machine-learning-based science [44]. These findings motivate the record-wise evaluation and the external evaluation adopted in this study.

## 2. Materials and Methods

### 2.1. Materials

In this study, we used the publicly available EEG Violence Dataset [13], which was also used in a later violence-detection study [45]. According to the dataset descriptions, the EEG signals were collected from 14 healthy university students aged 18–25 years with no reported history of neurological or psychiatric disorders [13,45].

During data acquisition, the participants watched violent video clips or meditation (relaxation) video clips, with clip durations between 1 and 5 min [13,45]; no further details of the stimuli or of the participants’ emotional responses are provided. The two classes are therefore defined by the stimulus condition: the neutral (control) class contains EEG recorded while meditation videos were viewed, and the violence class contains EEG recorded while violent videos were viewed. These conditions correspond to the affective states that the stimuli are intended to induce: a negative, high-arousal state elicited by violent content and a neutral, relaxed state elicited by meditation content. As is common for affective EEG datasets in which the labels are derived from the stimulus category, the labels reflect the intended emotional induction rather than individual self-reports. We use the labels “neutral (control)” and “violence” consistently in the text, tables and figures.

**Figure 2:**
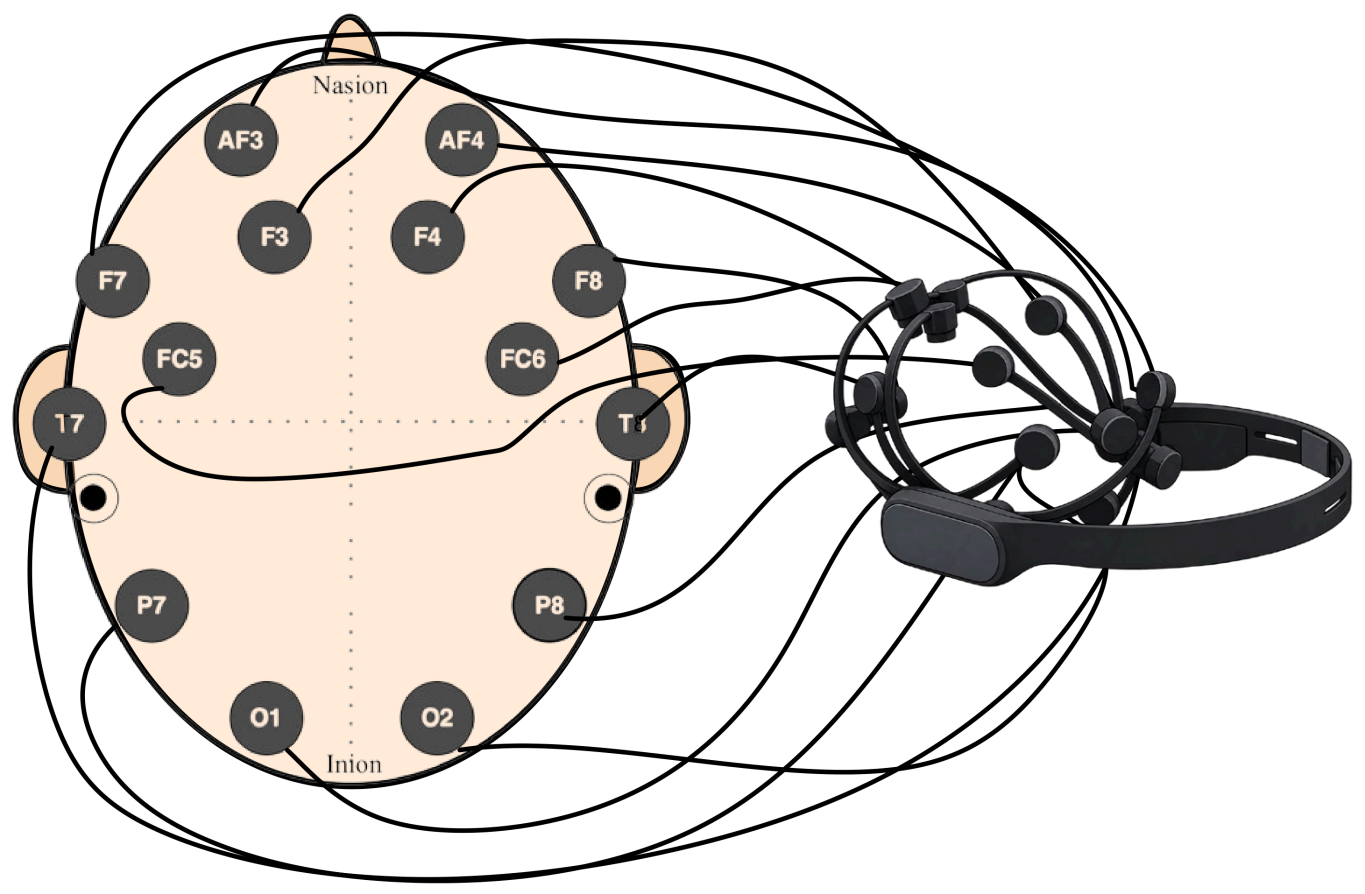
Emotiv EPOC X brain cap electrode placements.

The EEG signals were recorded using an Emotiv EPOC X brain cap (Emotiv Inc., San Francisco, CA, USA) with 14 channels. The sampling rate of the signals is 128 Hz. The headset uses saline-soaked felt electrodes with CMS/DRL reference electrodes; signals are internally sampled at 2048 Hz and down-sampled to 128 Hz, with a bandwidth of 0.16–43 Hz, 16-bit resolution and built-in 60 Hz notch filtering. The released dataset contains 728 non-overlapping 15-s segments (1920 samples × 14 channels) cut from consecutive time sections of 85 records (38 neutral and 47 violent-video records with 442 and 286 segments, respectively). Every record belongs to a single class, and the mapping between records and the 14 participants is not included in the public release, so a participant-wise evaluation cannot be constructed from the released data. Figure 2 shows the electrode placements of the 14-channel Emotiv brain cap according to the international 10–20 system.

The composition of the dataset is summarized in Table 1, and the class and number of segments of every record are listed in Appendix A. Because the segments of a record are consecutive sections of the same recording, they share the participant, the session and the electrode contact and are therefore not independent observations. All evaluations in this study were therefore performed record-wise: in LORO cross-validation, all segments of one record form the test set, and no segment of the test record is used for training, early stopping or the selection of the training hyperparameters.

The 14 active channels positioned to receive signals from different cortical regions of the brain are shown explicitly. There are 8 sensors for frontal region (AF3, AF4, F3, F4, F7, F8, FC5, FC6), 2 sensors for temporal region (T7, T8), 2 sensors for parietal region (P7, P8) and 2 sensors for occipital region (O1, O2). This standardized sensor configuration enables the simultaneous mapping of electrical activity in multiple brain regions accurately. The brain regions, shown in different colors, perform specialized emotional and cognitive processes. The frontal region performs complex mental operations such as emotion regulation, decision making and management of reactions to stress. The green-colored temporal regions on either side are responsible for analyzing auditory processes and emotional memory. The parietal region combines the information collected from several senses while the red colored occipital region takes part in the primary processing of visual stimuli. Because the frontal region is involved in the regulation of emotional responses, the densely placed frontal electrodes can be expected to provide discriminative information about the affective responses elicited by the violent and neutral videos.

**Figure 3:**
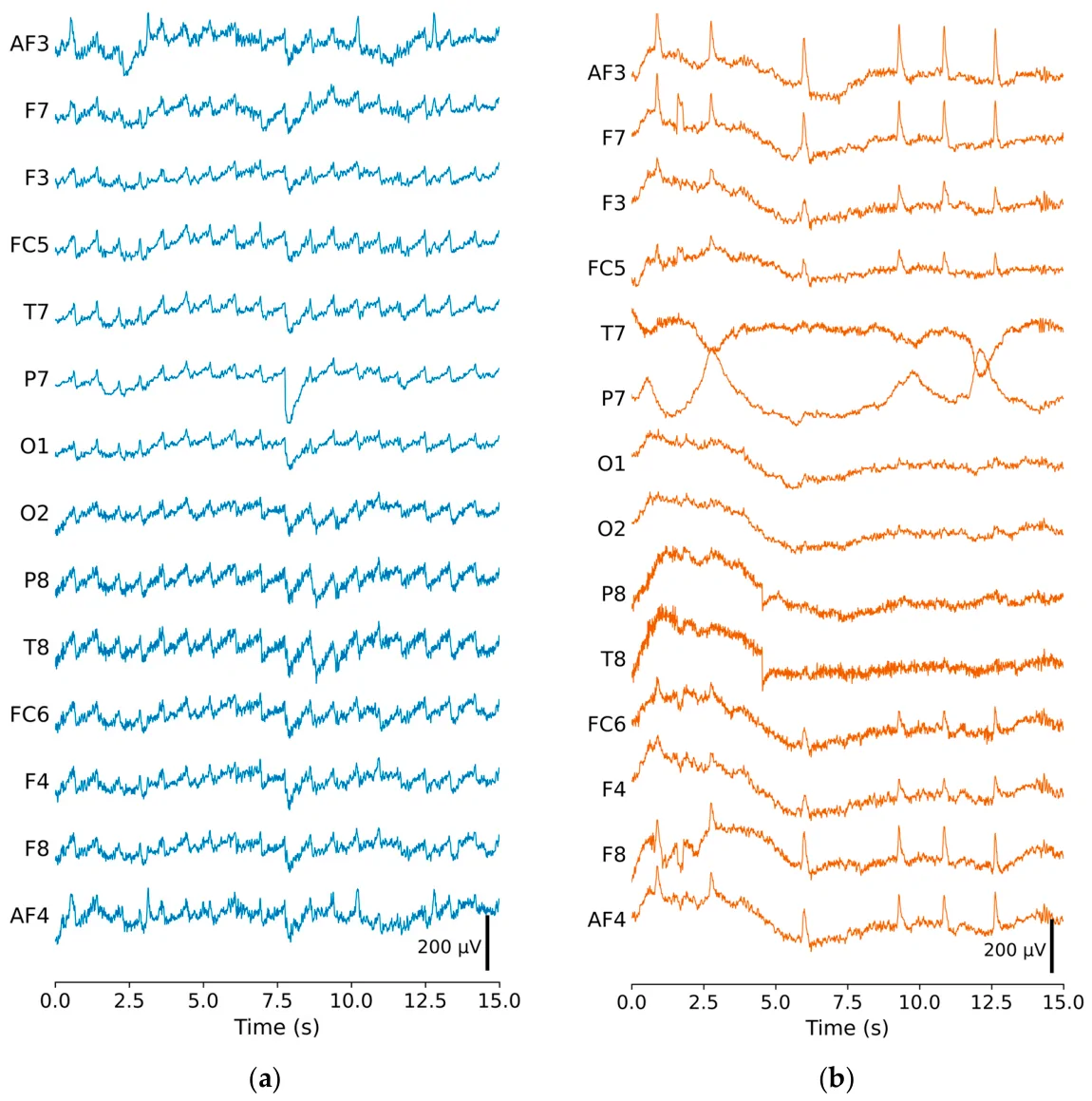
Example 15-s segments (14 channels, per-channel mean removed) from (**a**) a neutral (control) record (record 1, segment 1) and (**b**) a violent-video record (record 26, segment 1). Vertical bar: 200 µV.

Figure 3 shows one example segment from each class after removal of the per-channel mean; because the channels are stacked without individual y-axes, the vertical bar indicates an amplitude of 200 µV. The examples illustrate that the raw consumer-grade recordings contain non-neural components: the violent-video example shows large, synchronous deflections in the frontal channels (AF3, F7, F8 and AF4) that are consistent with eye blinks, and a step-like electrode artifact in P8 and T8. Single examples cannot be used to infer class differences; they are shown only to illustrate the signal quality.

**Figure 4:**
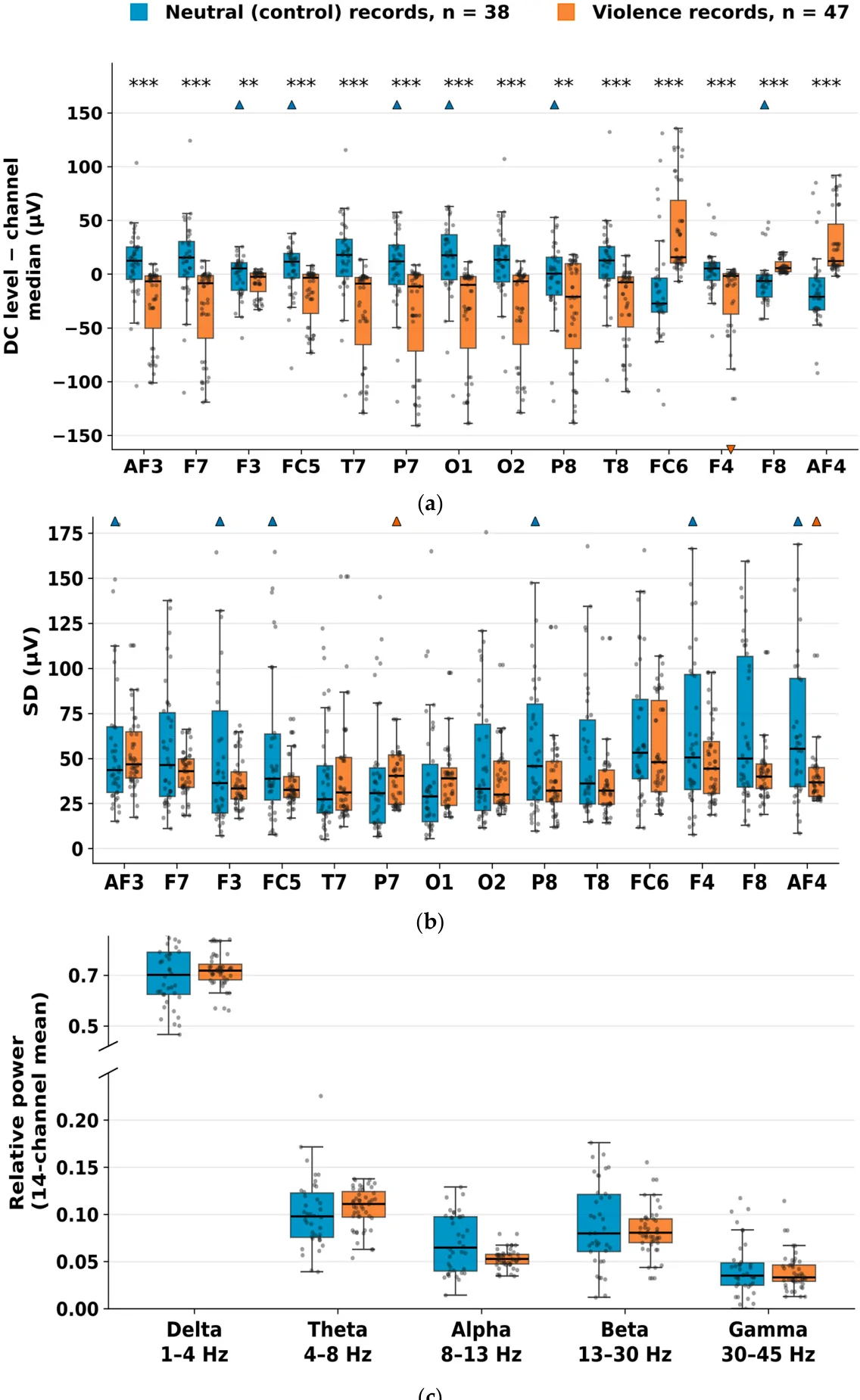
Record-level channel statistics of the two classes (728 segments; 38 neutral and 47 violence records): (**a**) electrode DC level relative to the channel median, (**b**) within-segment standard deviation after DC removal, and (**c**) channel-averaged relative band power. Two-sided Mann–Whitney U tests with Benjamini–Hochberg correction: ** q < 0.01, *** q < 0.001. Triangles at the axis limits mark values outside the displayed range.

To characterize the two classes quantitatively without the bias introduced by correlated segments, channel-wise statistics were computed at the record level (each of the 38 neutral and 47 violence records contributes one value, averaged over its segments) and compared with two-sided Mann–Whitney U tests, controlling the false discovery rate (FDR) across channels or bands with the Benjamini–Hochberg procedure as shown in Figure 4. In Figure 4, asterisks mark significant differences; values outside the displayed range are shown as triangles at the axis limits, and the *y*-axis of Figure 4c is interrupted between 0.25 and 0.42. The electrode DC level differed between the classes in all 14 channels (q < 0.05; Figure 4a), although it carries no oscillatory EEG information and mainly reflects the electrode–skin contact and the device offset. In contrast, neither the signal amplitude after DC removal (Figure 4b; all q > 0.11) nor the channel-averaged relative band power (Figure 4c; all q > 0.22) differed significantly between the classes. Channel-specific differences that appear when the segments are pooled (e.g., a higher variability at P7) are therefore not supported at the record level, and we refrain from interpreting channel-level differences as neural responses. Although all recordings were acquired with the same device and protocol [13,45], the systematic DC-level difference shows that the records of the two classes also differ in session- and participant-specific signal properties, such as the electrode–skin contact, which may arise because the two classes were recorded in different sessions or from different participants [13]; this is a confound that a classifier can exploit, and it is examined explicitly in the experiments.

In this work, the deep models receive the raw EEG segments. The only preprocessing step is Z-score normalization applied per channel within each segment, which also removes the electrode DC level. No band-pass filtering or artifact rejection is applied, so the models are exposed to the ocular, muscular and movement artifacts that are present in consumer-grade recordings; the implications of this choice are discussed in Section 4. Whether these artifacts differ between the two classes and influence the classification is analyzed in Section 3.5.

### 2.2. The Proposed Method

**Figure 5:**
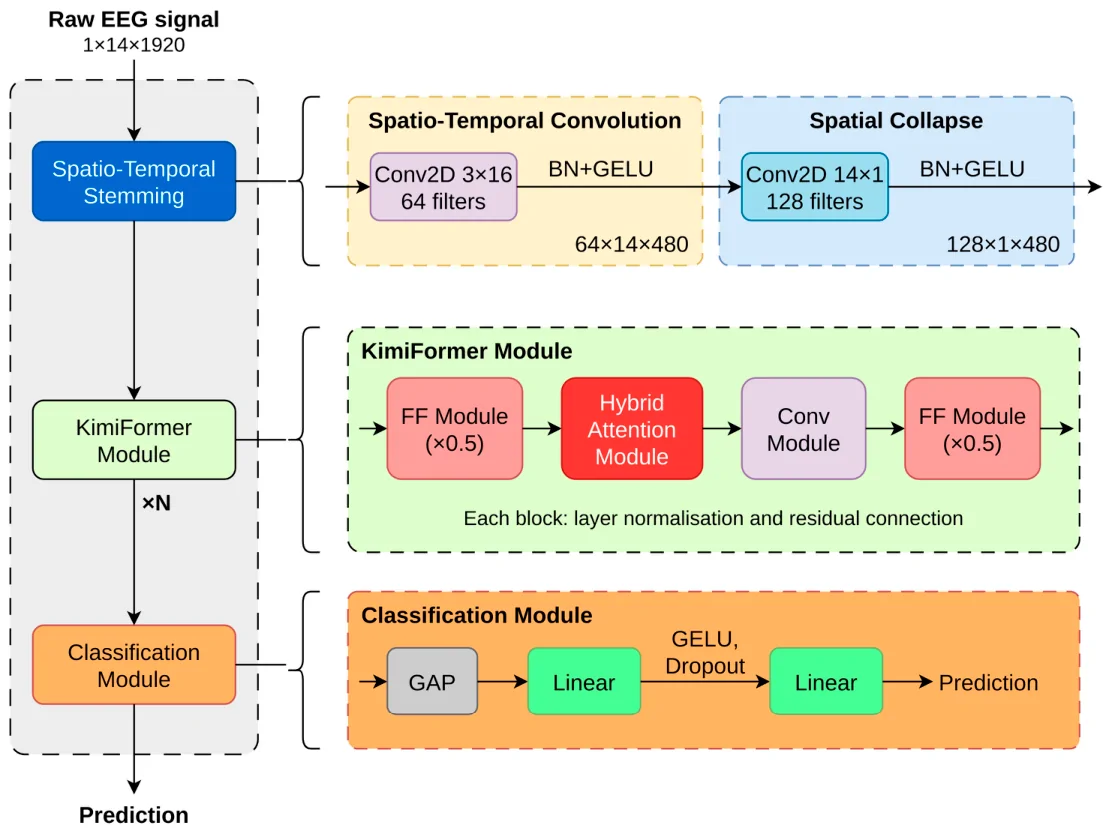
Graphical representation of the proposed KimiFormer model.

In this paper, we propose a Conformer-based hybrid attention model called KimiFormer. The KimiFormer model has feed-forward (FF) blocks, convolution blocks, and attention blocks like the original Conformer model. However, instead of using full multi-head self-attention in every layer, KimiFormer uses linear attention layers to obtain a more efficient and faster computation while reducing memory usage. The proposed model consists of three main modules. These are the STS module, the proposed KimiFormer module and the classification module. The STS module is used for extracting rich feature representations directly from EEG signals. The proposed STS module captures local temporal dependencies and spatial correlations simultaneously by using a hierarchical 2D convolution structure. This initial module transforms raw EEG signals into a compact embedding space and enables the KimiFormer model to learn high level feature representations effectively in the subsequent KimiFormer encoder. The KimiFormer block is the core module of the proposed architecture. It employs linear layers, attention layers and convolution layers in a Macaron-style arrangement to capture both global and local dependencies. The graphical representation of the KimiFormer model is given in Figure 5. As shown in Figure 5, the KimiFormer module consists of FF, attention, and convolution modules supported by residual connections and arranged sequentially. The modules of the proposed model are explained in detail in this section.

#### 2.2.1. STS Module

**Figure 6:**
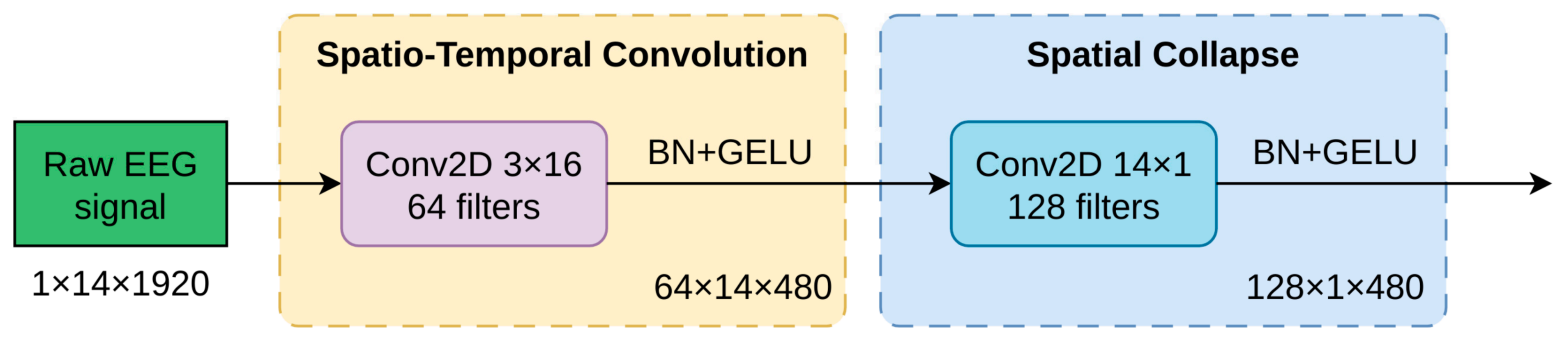
Architecture of the proposed STS module.

The input layer of the proposed model is the STS module. The STS module consists of the initial feature extraction layers. These layers are designed to accurately extract both temporal and spatial features from the raw signals. Traditional methods generally process the EEG signals as independent time series or flatten all channels of EEG signals before processing. In contrast, the proposed method aims to detect temporal and spatial relationships together to generate efficient and effective features. The STS module transforms the raw multichannel EEG signal into a high-level single channel representation. The graphical diagram of the STS module is shown in Figure 6.

The proposed STS module processes raw EEG recordings using a two-stage convolutional feature extractor, namely spatio-temporal convolution (STC) and spatial collapse (SC). These stages are designed to learn both local spatio-temporal features and global spatial representations.

The STC stage receives raw EEG signals with a shape of 1 × 14 × 1920 (channels × electrodes × time). The electrodes are the different channels of the brain signals. A two-dimensional convolution with a kernel size of 3 × 16 and 64 output feature maps is applied to jointly capture short-range temporal dynamics and local spatial correlations across adjacent electrodes. Using a kernel size of 3 in the spatial dimension enables the convolutional layer to jointly process signals from three adjacent electrodes. This allows the model to learn local spatial relationships between neighboring channels. The kernel size of 16 in the temporal dimension means the kernel processes the signal in 125 ms windows, as the signal sampling rate of the EEG signals is 128 Hz. This operation reduces the temporal dimension while preserving the full spatial resolution of the 14 electrodes. We used a non-symmetrical stride method in the STC stage, which is 1 in the spatial dimension and 4 in the temporal dimension. Zero padding of (1, 7) is applied so that the output keeps 14 electrodes and 480 time steps. The convolution output is followed by batch normalization (BN) and a Gaussian error linear unit (GELU) activation function to stabilize training and improve representational capacity. The transition table of the STS module is given in Table 2.

The SC stage receives the output obtained from the STC stage and applies a convolution with a kernel size of 14 × 1 and 128 output channels. This kernel spans the entire electrode dimension, effectively collapsing the multichannel spatial structure into a single spatial unit. The temporal resolution is preserved while the feature dimensionality increases, resulting in an output of 128 × 1 × 480. Similar to the previous stage, BN and GELU activation are applied to the convolution outputs.

#### 2.2.2. KimiFormer Module

**Figure 7:**
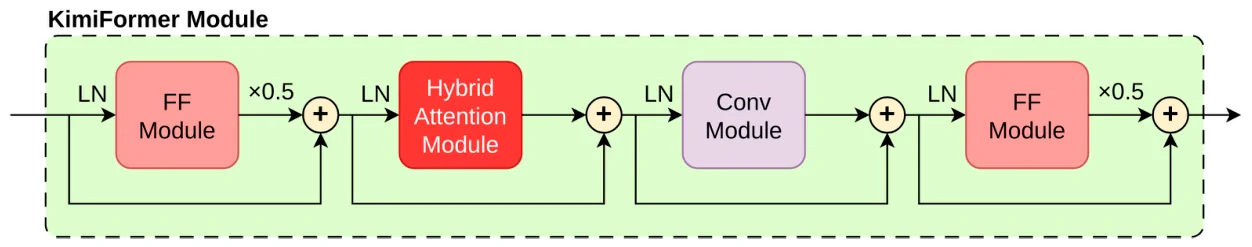
Graphical representation of the KimiFormer module.

In the proposed model, the structure of the KimiFormer module is inspired by the Conformer and Kimi Linear models. Each block follows a Macaron-style structure comprising two FF modules, an attention module, and a convolutional module, each preceded by layer normalization (LN) and followed by a residual connection. The graphical representation of the KimiFormer module is given in Figure 7.

Hybrid Attention Module: In long time series such as EEG signals, using the Full Self-Attention mechanism, which has standard *O*(*N*^2^) complexity, in every layer is costly in terms of memory and computation power. In comparison, while Linear Attention mechanisms provide efficiency, they can sometimes be insufficient in capturing complex, detailed relationships between signals [46]. The attention module has two different attention mechanisms in the KimiFormer model. We used linear attention and full multihead attention layers together. Linear attention layers are the default attention technique in our model since they have *O*(*N*) time complexity. In [47], the authors reported that combining linear and multi-head attention at a ratio of 3:1 gives the best results. For this reason, we used a multihead attention layer in every 4th attention layer. In the remaining layers, we used a linear attention layer whose implementation is based on the Flash Linear Attention (FLA) Python library. This design makes the model faster and less memory-intensive than full attention in every layer.

**Figure 8:**
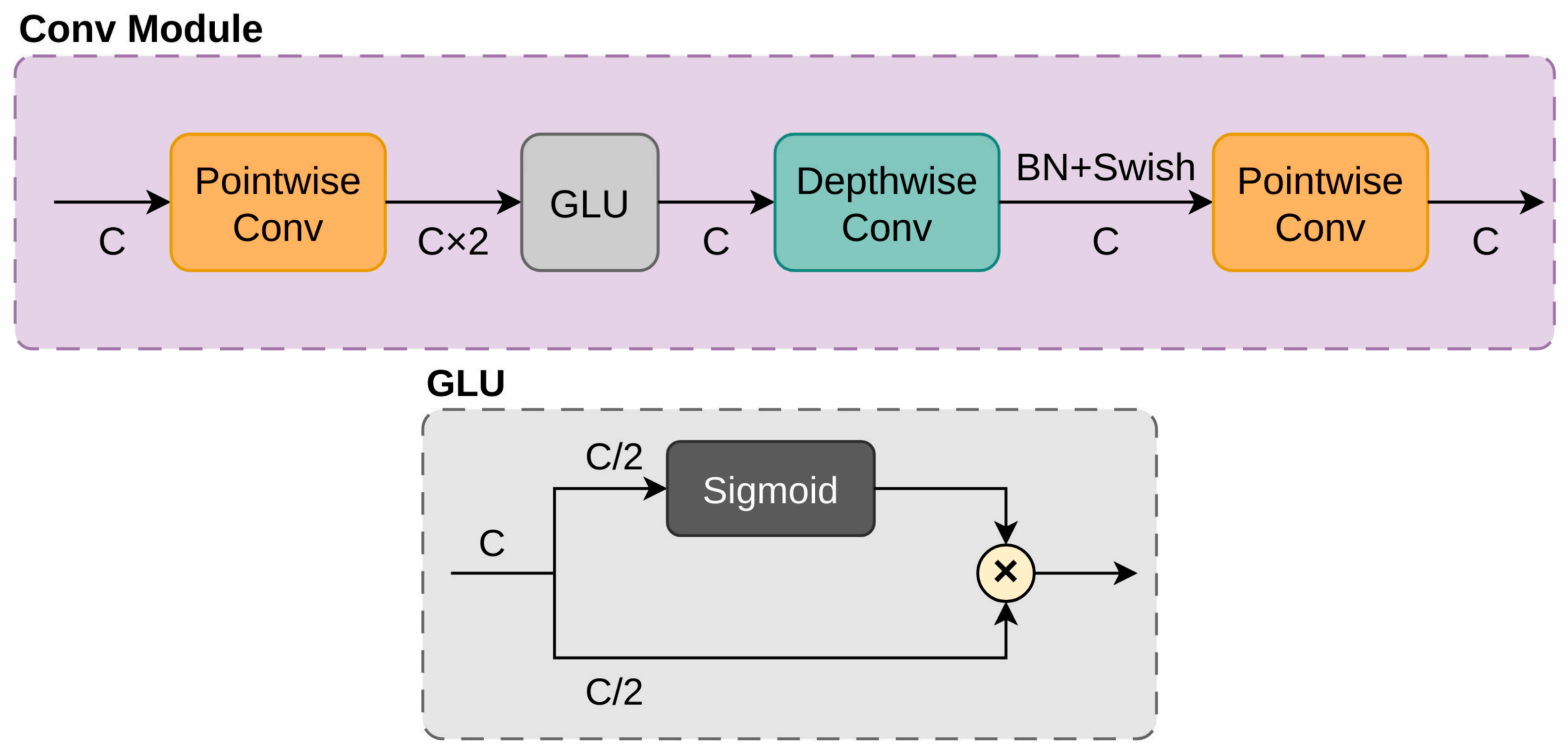
Architecture of the convolution module. The × symbol denotes element-wise multiplication.

Convolution Module: While attention mechanisms are successful at capturing long-term relationships, EEG signals are rich in short-term frequency patterns. KimiFormer uses a Convolution Module immediately after the Attention layer to capture these local features, as shown in Figure 8. In the Convolution module, pointwise convolution, gated linear unit (GLU) activation and depthwise convolution layers are performed sequentially followed by Swish activation. The pointwise convolution layer doubles the channel size of the input. The resulting output is fed into the GLU activation function. GLU splits the data into two equal parts (*A* and *B*); *A* is the data itself, while *B* is a gating mechanism since it passes through the sigmoid function and is multiplied by *A*. The mathematical formula of the GLU is given in Equation (1).

*GLU*(*A*, *B*) = *A* ⊗ *σ*(*B*) (1)

Here, ⊗ represents pointwise multiplication, and *σ* represents the Sigmoid function. This structure enables the model to learn which information should be passed to the next layer and which should be filtered out. This is particularly effective in eliminating noise in EEG signals.

A 1D depthwise convolution is applied on the features obtained from GLU, performing channel-based operations. This layer captures local temporal patterns while preserving spatial features. The final layer of the Convolution Module is a 1 × 1 pointwise convolution layer which computes the final importance of the features followed by a dropout layer. The transition table of the Convolution module is given in Table 3.

As can be seen from the transition table, the convolution module first increases the number of channels with 1 × 1 pointwise convolutions and then uses GLU to filter information flow. Then, local temporal features are obtained by applying depthwise convolution with a 31-dimensional kernel. Unlike the STS module, in this module the stride is fixed at 1 (S = 1) for all convolution operations, thus preserving the temporal resolution of the input (T = 480) and targeting only contextual enrichment.

**Figure 9:**
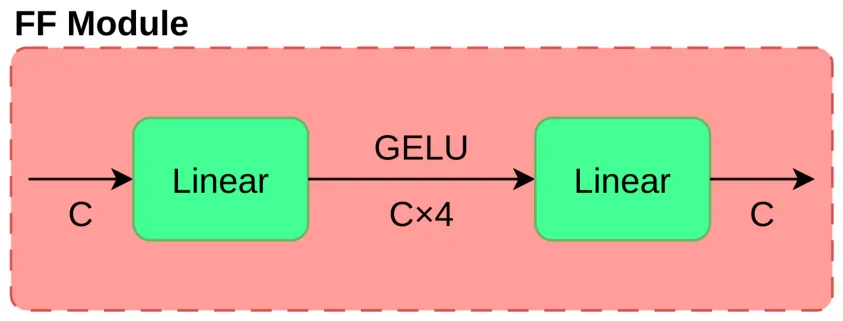
The structure of the FF Module.

Macaron-Style Feed-Forward Module: FF modules are located at the beginning and end stages of the KimiFormer block. Attention and convolutional modules are located between the two FF modules. This structure is called the Macaron Net architecture [48]. Furthermore, in terms of the change in the model’s representation capacity, this layer forms an inverted bottleneck structure. The expansion ratio of the module has been set to 4 in this study. The structure of the FF module is given in Figure 9.

The narrow-wide-narrow structure ensures that the model maintains its parameter efficiency while creating a much richer feature map compared to standard layers. To preserve variance in the signal flow and ensure gradient stability, these modules are integrated using the half-step strategy as shown in [48]. This scaling process prevents signal magnitudes from increasing uncontrollably as the model depth increases and provides more stable convergence during the optimization process. The transition table of the module is given in Table 4.

#### 2.2.3. Classification Module

**Figure 10:**
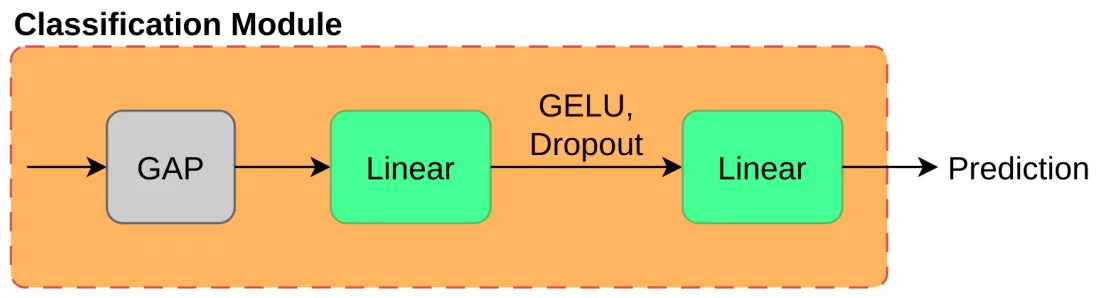
Graphical representation of the classification module.

The last phase of the KimiFormer model is the classification module. In this module, rich context feature maps are transformed into class predictions. The features obtained in the KimiFormer modules are first flattened using a global average pooling (GAP) layer to achieve invariance against shifts in the time axis and reduce the risk of overfitting. After that, the flattened features are fed into two consecutive linear layers separated by GELU activation and dropout layers. The SoftMax activation function is used after the last linear layer to generate class probabilities. The structure of the classification module is given in Figure 10.

The mathematical representation of the layer transitions of the KimiFormer model is given below:

Step 1: Transform EEG signals to rich context embeddings with STS module.

(2) $$H_{1}=\varrho (\mathrm{BN}(C_{64,s:(1,4)}^{\left(3,16\right)}(X))$$

(3) $$Z_{0}=\mathrm{Permute}(\varrho (\mathrm{BN}(C_{128,s:(1,1)}^{\left(14,1\right)}(H_{1}))))$$

Herein *C* represents convolution, *BN* represents batch normalization and *ϱ* represents GELU activation layers. *X* and *Z*_0_ represent the input EEG signals and the rich context embeddings obtained from the raw signals.

Step 2: Process embeddings through the KimiFormer main block to extract features from the input embeddings. This block is repeated *N* times, where *N* = 8 in our experiments.

- Phase 1: Apply an inverted bottleneck and half-step FF module in the FFModule to enrich the feature space.

(4) $${\bar{Z}}_{n}=Z_{n-1}+\frac{1}{2}\mathrm{FFModule}(\mathrm{LN}(Z_{n-1}))$$

*FFModule*(*x*) = *W*_2_.(*ϱ*(*W*_1_.*x* + *b*_1_)) + *b*_2_ (5)

Here *W*_1_ and *W*_2_ denote weight matrices of the linear layers in FFModule. The first layer expands the feature dimension by a factor of four, while the second layer projects the expanded representation back to the original feature dimension. LN denotes layer normalization.

- Phase 2: Process the rich feature space in hybrid attention blocks to extract long-range dependencies.

(6) $$A_{n}=\left\{\mathrm{FullAttn}\left(\mathrm{LN}\left({\bar{Z}}_{n}\right)\right),\mathrm{if}\;n\left(\mathrm{mod}4\right)=0,\mathrm{LinearAttn}\left(\mathrm{LN}\left({\bar{Z}}_{n}\right)\right),\mathrm{else}\right\}\;Z_{n}^{\prime }=Z_{n}+A_{n}$$

Here, *n* indicates which step of the main block is performed. The attention mechanism applied at each stage is determined by the step index *n*.

- Phase 3: Apply ConvModule to extract local dependencies.

(7) $$Z_{n}^{\prime \prime }=Z_{n}^{\prime }+\mathrm{ConvMod}(\mathrm{LN}(Z_{n}^{\prime }))$$

*ConvMod*(*X*) = *Pointwise*(*Swish*(*Depthwise*(*GLU*(*Pointwise*(*X*))))) (8)

- Phase 4: Repeat phase 1 to obtain the final features.

(9) $$Z_{n}=Z_{n}^{\prime \prime }+\frac{1}{2}\mathrm{FFModule}(\mathrm{LN}(Z_{n}^{\prime \prime }))$$

Step 3: Transform the output of the last encoder block into class predictions.

(10) $$v=\mathrm{GAP}(Z_{N})=\frac{1}{T^{\prime }}\sum_{t=1}^{T^{\prime }}Z_{N}\left[t\right]$$

(11) $$\hat{y}=\mathrm{Softmax}(W_{\mathrm{fc}2}.{\varrho (W}_{\mathrm{fc}1}.v+b_{\mathrm{fc}1}){+b}_{\mathrm{fc}2})$$

Herein, GAP denotes the global average pooling layer, *T*′ represents the sequence length of the final feature tensor that is output from the encoder blocks.

## 3. Results

In this section, we present the experimental results and parameter details of the proposed KimiFormer model. The proposed model is inspired by architectures like ConvNext [49], Conformer [50], and Kimi Linear [47]. The proposed model uses raw EEG signals to transform them into rich context representations. Then it captures the long-context dependencies using hybrid attention blocks. Finally, the resulting features are classified by the classification head. The classification performance, stability, and computational efficiency of the model are comprehensively analyzed. To assess the model’s generalization ability and the risk of overfitting, the experiments are carried out focusing on two different validation strategies: (1) Testing the model’s robustness with segment-wise 10-fold and record-wise LORO cross-validation, and (2) Comparative analysis with other modern architectures and baseline models in the literature.

### 3.1. Experimental Setup and Training Parameters

The proposed model was implemented in Python 3.13.16. We used PyTorch 2.11.0 [51] and FLA 0.5.2 [52] libraries to develop deep neural network architectures, and scikit-learn 1.6.1 for the cross-validation splits and the feature-based baseline classifiers. We conducted all the experiments on a workstation equipped with 32 GB of RAM and an NVIDIA Tesla T4 GPU (NVIDIA Corporation, Santa Clara, CA, USA) with 16 GB of memory. The hyperparameters of the proposed model are given in Table 5.

The model was trained using the AdamW optimizer, which improves the generalization performance of adaptive algorithms on CNNs and ViTs [53]. The initial learning rate was set to 1 × 10^−4^ and the weight decay coefficient to 0.01. Training was performed with a batch size of 32 for a maximum of 50 epochs. Early stopping (patience = 10 epochs) monitored the cross-entropy loss on an inner validation set carved from the training part of each fold only (20% of the training segments for segment-wise cross-validation and 20% of the training records for record-wise cross-validation), and the weights with the lowest inner-validation loss were restored. The held-out test fold was never used for training or early stopping and was evaluated once; the selection of the hyperparameters is described below. The cross-entropy loss function was used to optimize the classification objective, which is appropriate for the binary classification setting considered in this study. We used full attention layers in every fourth layer of attention, and linear attention layers in all other attention layers to obtain a more efficient model architecture. During model development, these settings were explored with a restricted grid search on a single random split of the segments (70% training, 20% validation and 10% test), with the weighted F1-score on the validation part as the selection criterion; the evaluated search space and the values chosen in this exploration are given in Table 6. Because this split was drawn from the same dataset, records that are held out in the record-wise evaluation could have contributed to this exploration. For the record-wise evaluation, the training hyperparameters were therefore selected again with a nested procedure in which every held-out record is excluded from the selection of these hyperparameters. In each of the 85 outer LORO folds, the inner validation set described above (20% of the training records) was used for selection as well as for early stopping: nine candidate configurations, i.e., all combinations of the learning rate (1 × 10^−3^, 1 × 10^−4^ and 1 × 10^−5^) and the batch size (16, 32 and 64), were trained on the remaining training records, and the candidate with the lowest inner-validation cross-entropy loss was used, without refitting, to classify the held-out record. All candidates used the same inner split, initialization and training loop, and the candidate list and the selection rule were fixed before the nested experiments were run. The architectural settings (the 3:1 ratio of linear to full attention, N = 8 blocks and a depthwise kernel size of 31) were kept at the values in Table 6 and were not included in this nested selection. To examine whether their non-nested choice affects the reported performance, the alternative numbers of blocks and kernel sizes in Table 6 and the linear-attention-only and full-attention-only variants were additionally evaluated under the same LORO splits, and the results are presented and discussed in Section 3.3 and Section 4.2 and in Appendix A (Table A2). The segment-wise 10-fold cross-validation, which is reported only for comparability with previous studies, uses the configuration in Table 5 and was not nested.

The selection of the depthwise convolution kernel size was determined through a combination of neurophysiological reasoning and the exploration on the development split described above. In the proposed architecture, the initial STS module reduces the temporal dimension of the 15-s raw EEG input from 1920 to 480 steps via a non-symmetrical stride. Consequently, each temporal step in the subsequent KimiFormer encoder corresponds to approximately 0.03125 s. By employing a kernel size of 31, the depthwise convolution spans a localized temporal receptive field of roughly 0.96 s (~1 s). This ~1 s window is well suited for EEG analysis, as it can capture at least one full cycle of low-frequency neural oscillations, which are critical biomarkers for cognitive stress and emotion regulation. On the development split, the smaller kernel (K = 15, ~0.5 s) and the shallower variants (N ∈ {4, 6}) yielded lower weighted F1-scores, whereas the larger kernel (K = 63, ~2 s) and the deeper variant (N = 10) did not improve it and increased the number of parameters and the inference latency; the values in Table 5 were therefore adopted. Under the record-wise LORO evaluation, however, the differences between these alternatives were small and not in favor of the adopted configuration: with the training hyperparameters of Table 5, the LORO accuracy was 96.8%, 96.3%, 95.7% and 95.1% for N = 4, 6, 8 and 10, and 96.0%, 95.7% and 95.3% for kernel sizes of 15, 31 and 63, respectively, with overlapping confidence intervals (Appendix A, Table A2). The adopted configuration is therefore not an optimum for this dataset, and its non-nested choice did not inflate the reported record-wise performance.

**Figure 11:**
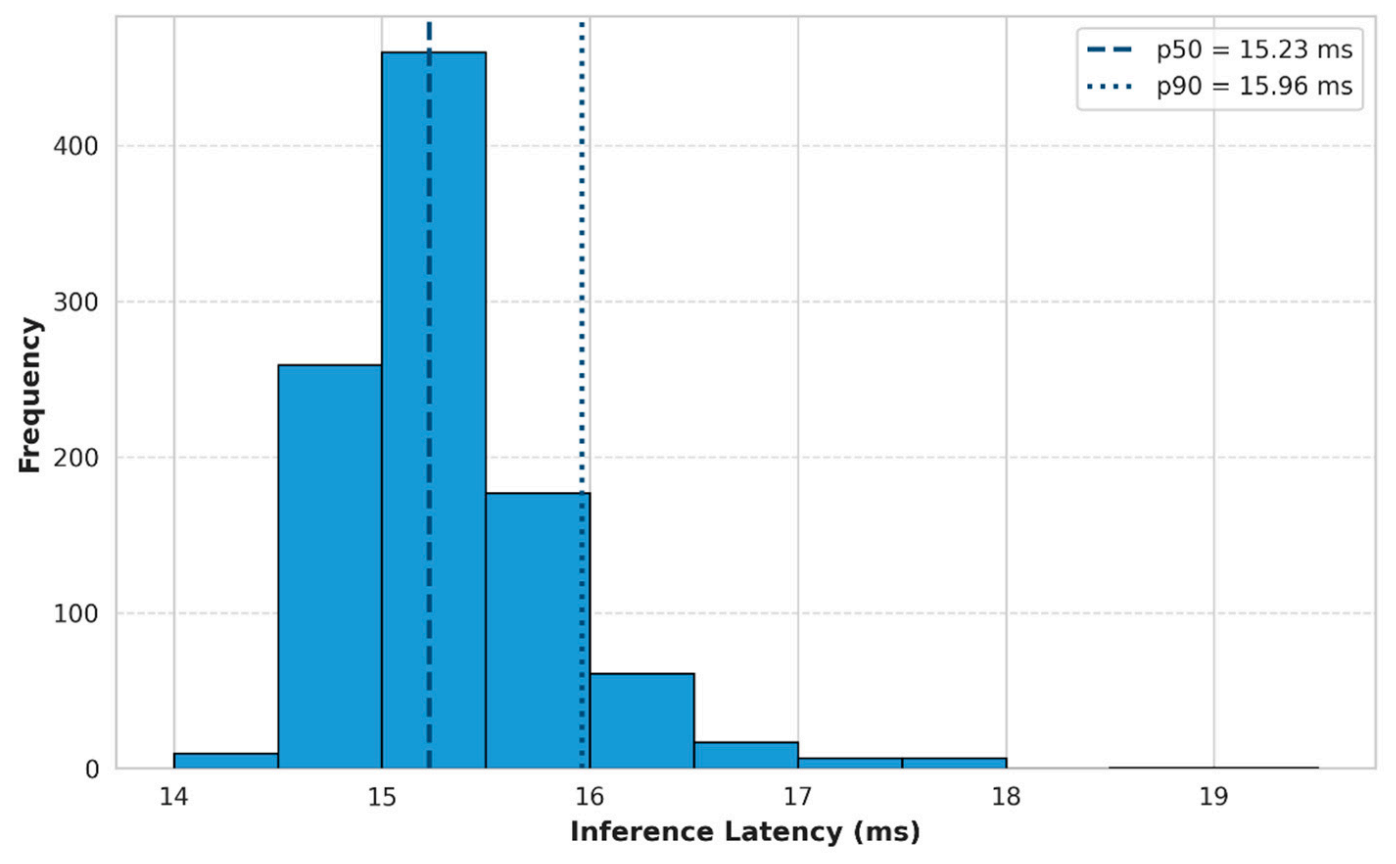
Distribution of inference latency measured on an NVIDIA Tesla T4 GPU with batch size 1. Dashed and dotted lines indicate the p50 and p90 percentiles, respectively. The results demonstrate stable and consistent inference latency across 1000 runs.

On an NVIDIA Tesla T4 GPU, the proposed model achieves an average inference latency of 15.64 ms per sample (batch size = 1), with p50/p90 latencies of 15.23/15.96 ms (~60 FPS). The latency was measured over 1000 runs on a sample input. The distribution of inference latency of the model is given in Figure 11. The model contains 3.2M parameters and requires 1.51 GFLOPs per forward pass. These results make the KimiFormer model suitable for real-time and resource-constrained applications.

### 3.2. Classification Performance

**Figure 12:**
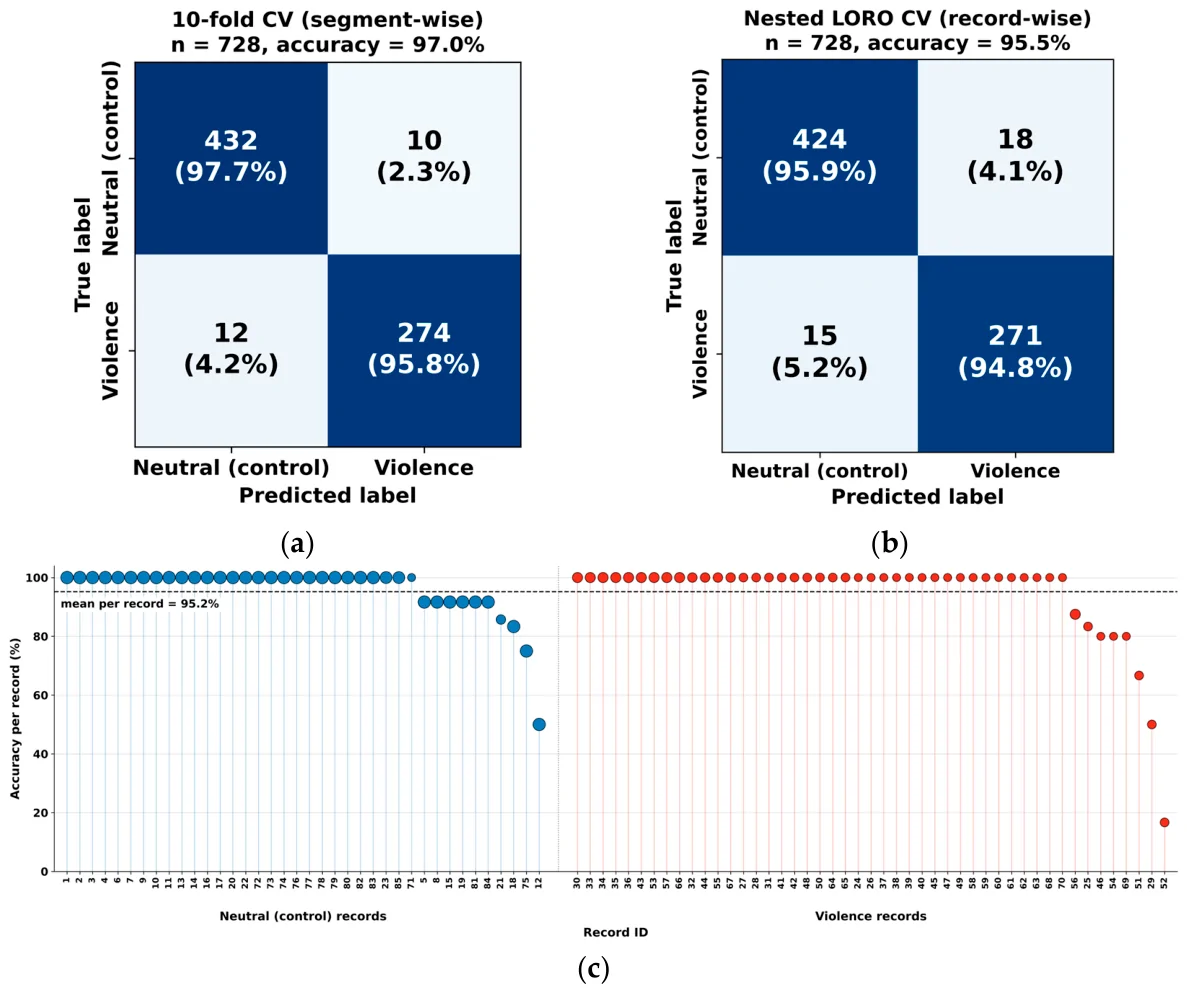
Classification results of the proposed model (728 segments): pooled confusion matrices under (**a**) segment-wise 10-fold and (**b**) record-wise LORO cross-validation with nested hyperparameter selection; (**c**) LORO accuracy of each of the 85 held-out records. In (**a**,**b**), darker shades of blue indicate higher percentages.

The proposed model was trained using the dataset explained in Section 2.1. We used widely adopted performance metrics to calculate the classification performance of the model. The metrics used in this study include accuracy, balanced accuracy, precision, recall (sensitivity), specificity, F1-score, weighted F1-score, the Matthews correlation coefficient (MCC) and the area under the ROC curve (AUC), with the violence class as the positive class. Balanced accuracy is the mean of sensitivity and specificity, i.e., the mean of the per-class recalls; it is reported in addition to accuracy because the classes are imbalanced (442 neutral and 286 violence segments), so that accuracy alone can be inflated by the majority class. The MCC is the correlation between the true and the predicted labels; it uses all four cells of the confusion matrix, ranges from −1 to 1 and is robust to class imbalance. These metrics highlight the performance of the proposed model in different aspects, such as overall correctness, class-wise prediction reliability, sensitivity to positive samples, and the balance between precision and recall. Each segment was classified once by a model that had not seen it during training, and the predictions of all folds were pooled. The proposed model achieved an overall classification accuracy of 97.0% under segment-wise 10-fold cross-validation and 95.5% under record-wise LORO cross-validation with nested hyperparameter selection. The corresponding pooled confusion matrices are shown in Figure 12.

**Figure 13:**
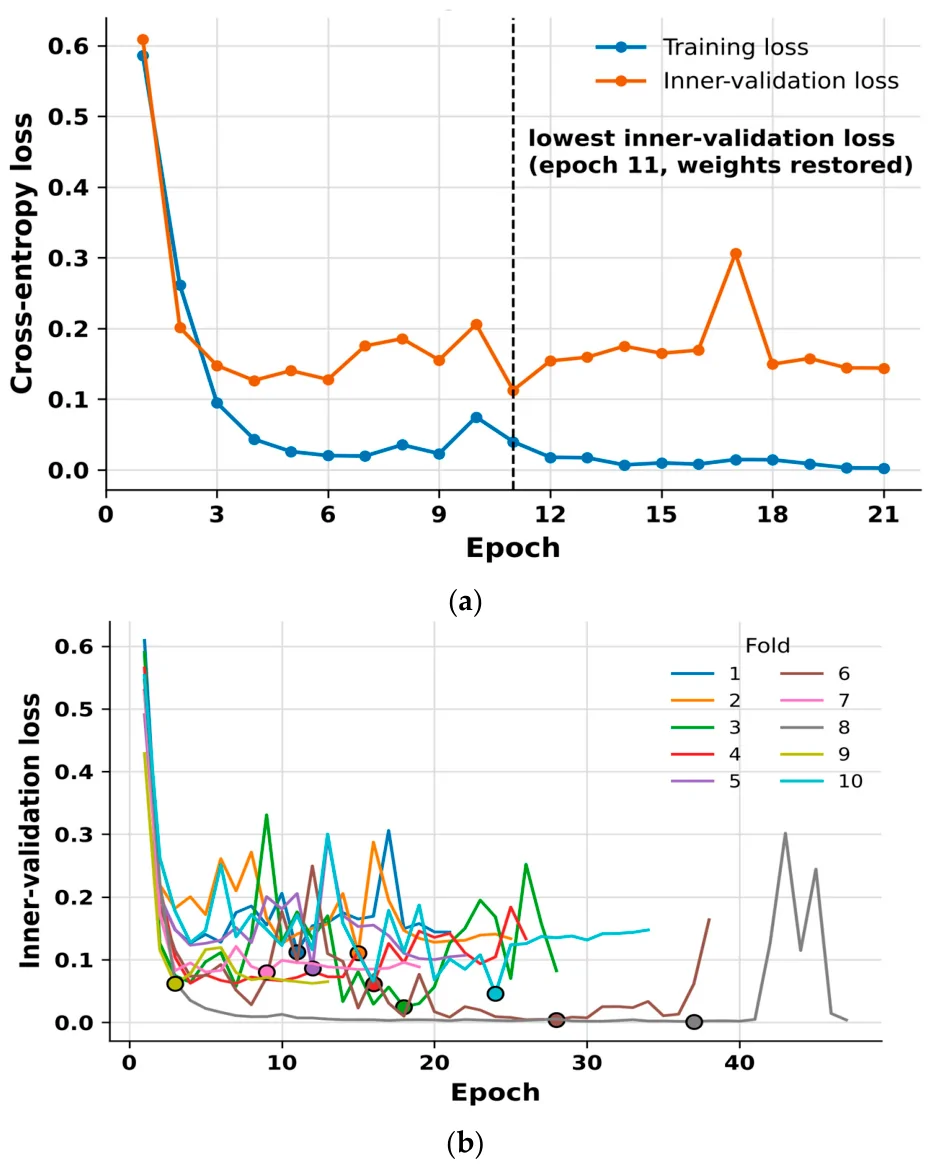
Training curves of the proposed model under 10-fold cross-validation: (**a**) training and inner-validation loss of the first fold; (**b**) inner-validation loss of all folds. The dashed vertical line in (**a**) marks the epoch with the lowest inner-validation loss, whose weights were restored.

As shown in Figure 12, both classes are recognized with similar reliability: 97.7% of the neutral (control) segments and 95.8% of the violence segments are classified correctly under 10-fold cross-validation, and 95.9% and 94.8%, respectively, under LORO cross-validation. Because the record-to-participant mapping is not part of the public release of the dataset, the variability between participants cannot be assessed directly; we therefore report the LORO result for each of the 85 held-out records in Figure 12c. Sixty-seven of the 85 records (79%) were classified without any error and 73 records (86%) with an accuracy of at least 90%; the mean per-record accuracy was 95.2% ± 13.0% (SD), with 95.9% ± 9.6% for the neutral (control) and 94.6% ± 15.3% for the violence records. When the segments of a record are combined by majority vote, 83 of the 85 records (97.6%) are assigned to the correct class (ties counted as errors); the two misassigned records are violence records 29 and 52. The lowest per-record accuracies were obtained for violence record 52 (one of six segments correct) and for violence record 29 and neutral (control) record 12 (50% each). The marker sizes in Figure 12c are proportional to the number of segments of the record (5–12), and the dashed line marks the mean per-record accuracy, in which every record has the same weight; the pooled accuracy over all 728 segments is 95.5%. To better understand the training process and to confirm that overfitting is controlled, the training curves are shown in Figure 13. Figure 13a shows the training loss and the inner-validation loss of the first fold of the 10-fold cross-validation, and Figure 13b shows the inner-validation loss of all ten folds. Markers in the graphics indicate the epoch with the lowest inner-validation loss, whose weights were restored.

The training loss decreases rapidly during the first epochs and approaches zero, whereas the inner-validation loss reaches its minimum after a few epochs (epoch 11 in the first fold) and then fluctuates around a low level. Because the weights of the epoch with the lowest inner-validation loss are restored and training stops when this loss has not improved for 10 epochs, the reported models are not taken from the final, over-trained epochs. The inner-validation loss curves of all folds (Figure 13b) show the same behavior, with minima between epochs 3 and 37, which indicates stable learning across different training partitions.

### 3.3. Cross-Validation Results

**Figure 14:**
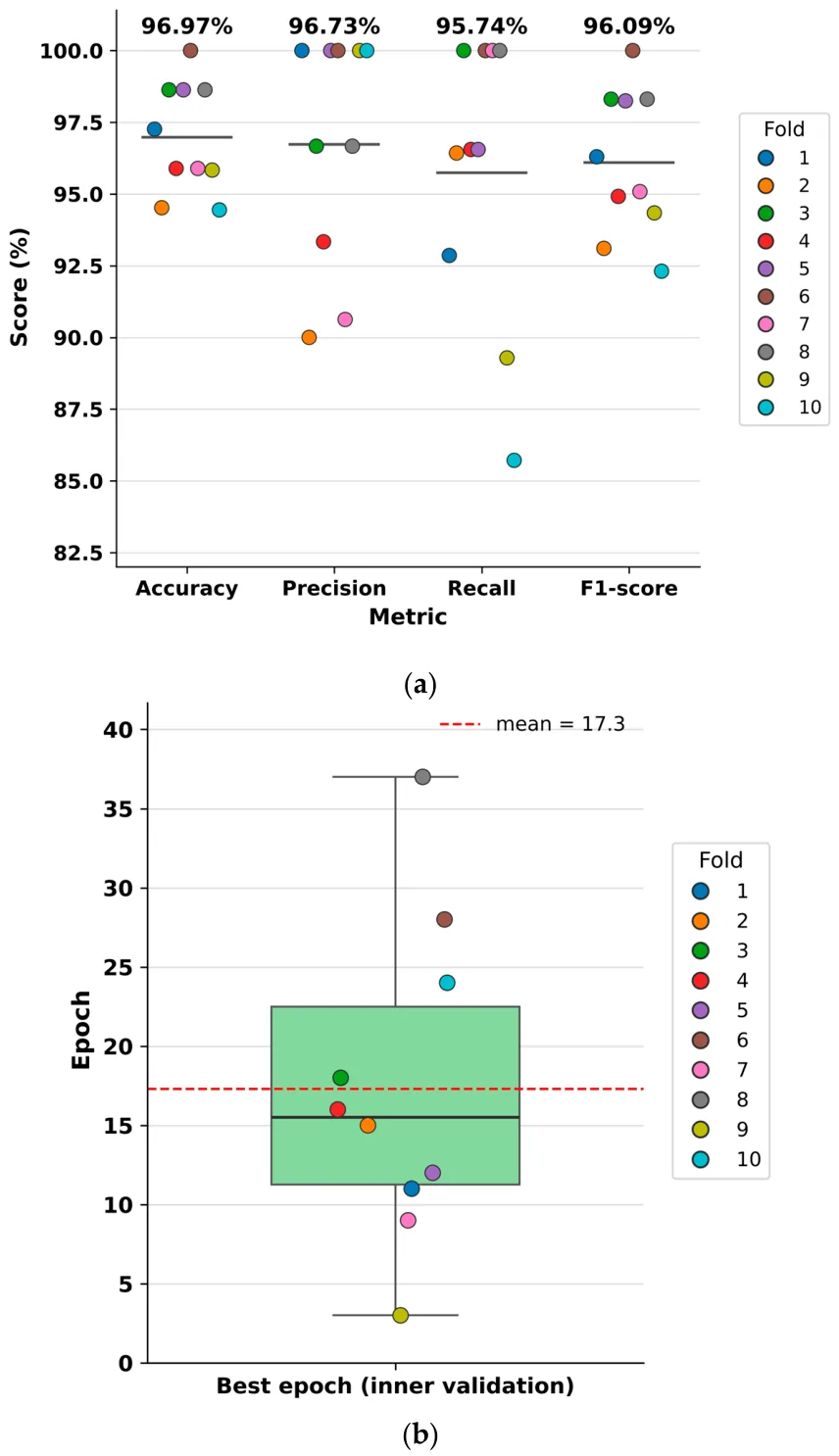
Per-fold results of the 10-fold cross-validation: (**a**) accuracy, precision, recall and F1-score of each fold (violence = positive class); (**b**) distribution of the epoch with the lowest inner-validation loss (early-stopping epoch); the dashed line marks the mean.

For 10-fold cross-validation, the 728 segments were randomly assigned to 10 stratified folds. Because consecutive segments of the same recording can therefore occur in both the training and the test folds, segment-wise 10-fold cross-validation does not measure subject- or recording-independent performance; it is reported only for comparability with previous studies on this dataset, and the record-wise LORO evaluation described below is the main evaluation of this study. Under this scheme, the model demonstrates consistently high performance: the per-fold accuracy ranged from 94.4% to 100% (mean 96.97%), which indicates stable behavior across different training and test partitions. The accuracy, precision, recall and F1-score obtained in each fold, along with the epoch selected by early stopping in each fold, are illustrated in Figure 14.

As seen in Figure 14, the model obtains consistently high performance across all folds. This means that the performance of the model does not depend on a specific partitioning of the data. The mean per-fold precision (96.73%) was slightly higher than the mean per-fold recall (95.74%), i.e., false alarms on neutral (control) segments were slightly less frequent than missed violence segments. The epochs selected by early stopping ranged from 3 to 37, with a median of 15.5 and a mean of 17.3 epochs. This variability suggests that while the model converges reliably, the optimal stopping point differs slightly across folds due to variations in training data composition. Overall, the epoch distribution indicates stable and efficient convergence without excessive training, supporting the reliability of the selected training configuration. We also evaluated the model with LORO cross-validation, in which all segments of one record form the test set and the inner validation set for early stopping is drawn from the training records only, so that no segment of the test record is used for training, early stopping or the selection of the training hyperparameters. The model also obtained robust classification results under LORO validation, with an accuracy of 95.5% and a balanced accuracy of 95.3%. The 95% confidence intervals, obtained with a bootstrap over records (2000 resamples), were 92.7–97.6% for accuracy, 92.4–97.6% for balanced accuracy, 89.8–98.3% for sensitivity (94.8%), 92.4–98.6% for specificity (95.9%) and 0.847–0.951 for the MCC (0.905) under LORO validation, and 95.5–98.3%, 95.0–98.3%, 92.7–98.6% (95.8%), 96.3–99.0% (97.7%) and 0.905–0.963 (MCC = 0.937), respectively, under 10-fold cross-validation; a majority-class classifier would obtain an accuracy of 60.7%, a balanced accuracy of 50% and an MCC of 0. Because every LORO test fold contains a single class, precision, recall and F1-score cannot be computed meaningfully per fold; all metrics are therefore computed from the pooled out-of-fold predictions of all folds. The obtained evaluation metrics are given in Table 7 for two different validation scenarios.

In the nested procedure, the selected candidate varied between the outer folds: a learning rate of 1 × 10^−3^ was selected in 50, 1 × 10^−4^ in 31 and 1 × 10^−5^ in 4 of the 85 folds, and a batch size of 16, 32 and 64 in 40, 27 and 18 folds, respectively. Nevertheless, the nested accuracy (95.5%) was almost identical to the accuracy obtained when the configuration in Table 5 was used in every fold (95.7%, 95% confidence interval (CI) 93.5–97.7%), i.e., 33 instead of 31 misclassified segments out of 728. When each of the nine candidates was used unchanged in all folds, the LORO accuracy ranged from 94.1% to 97.0% (Appendix A, Table A2), and the configuration in Table 5 ranked third. These results are reported as a sensitivity analysis only, because choosing the best of them would again use the test records for model selection; the nested result is the performance estimate of this study. They show that the record-wise performance of KimiFormer depends little on the training hyperparameters and that the earlier, non-nested choice of these hyperparameters did not inflate the reported performance. The architectural settings, which were not part of the nested selection, were examined in the same way: when the number of blocks or the kernel size was varied with the training hyperparameters of Table 5, the LORO accuracy ranged from 95.1% (N = 10) to 96.8% (N = 4), and the adopted configuration (95.7%) was again not the best of these alternatives (Appendix A, Table A2). The reported performance therefore does not depend on a favorable, test-informed choice of the architecture; the attention variants are discussed in Section 4.2.

To assess the difficulty of the dataset and to compare KimiFormer with conventional feature-based classifiers, we evaluated five baselines: logistic regression, KNN (k = 5), linear discriminant analysis, an SVM with a radial basis function (RBF) kernel and RF. Their input consisted of hand-crafted features commonly used in EEG classification, namely the minimum, maximum and standard deviation of every channel and the absolute power of every channel in the delta (0.5–4 Hz), theta (4–8 Hz), alpha (8–13 Hz), beta (13–30 Hz) and gamma (30–64 Hz) bands (Welch method, 2-s windows), i.e., 112 features per segment. The features were computed from the same z-score-normalized segments that form the input of KimiFormer, so that the baselines and KimiFormer received exactly the same information, and were standardized within each training fold. All baselines were evaluated with exactly the same segment-wise 10-fold and record-wise LORO splits as KimiFormer, and their metrics were computed from the pooled out-of-fold predictions in the same way.

Under record-wise LORO validation, KimiFormer obtained the highest accuracy (95.5%), balanced accuracy (95.3%), F1-score and MCC (0.905) of all models. It clearly outperformed the linear and distance-based classifiers, i.e., logistic regression (90.1%), linear discriminant analysis (87.9%) and KNN (87.9%), as well as the SVM (93.1%), and it also exceeded the strongest baseline, RF (94.8%), with 33 instead of 38 misclassified segments out of 728. KimiFormer and RF produced the same number of false alarms on neutral (control) recordings under LORO validation (18 of 442 segments; specificity 95.9%), whereas the AUC of RF (0.985) was similar to that of KimiFormer (0.981). Under segment-wise 10-fold validation, KimiFormer (97.0%) was likewise more accurate than all baselines (93.8–96.7%). The good performance of RF and SVM shows that the two conditions of this dataset can be separated with simple spectral and waveform statistics, in agreement with the very high accuracies reported for feature-engineering pipelines on the same data [13,45]. In contrast to these pipelines, KimiFormer reaches a higher level without any hand-crafted feature extraction or feature selection: it learns directly from the raw multichannel signal, so that the same architecture can be applied to other recordings and tasks without redesigning the features. Moreover, KimiFormer showed the smallest decrease of all models when moving from segment-wise to record-wise evaluation, indicating that its performance depends least on segments of the test recording being present in the training set. Because this dataset alone cannot discriminate between strong models, we additionally evaluated KimiFormer on the more challenging DREAMER benchmark, in which every participant experienced all conditions; there, under an identical subject-independent protocol, KimiFormer and the established raw-EEG architectures EEGNet and EEG-Conformer all performed close to chance; KimiFormer obtained numerically slightly higher values, but the differences were not statistically significant.

In conclusion, the results demonstrate that the proposed model achieves robust and consistent performance under both segment-wise 10-fold and record-wise LORO cross-validation. When all segments of the test record were excluded from training, the accuracy decreased by only 1.5 percentage points (from 97.0% to 95.5%), which indicates that the performance of the model does not depend on adjacent segments of the same recording being present in the training set.

### 3.4. Subject-Independent Evaluation on the DREAMER Dataset

To evaluate the proposed model on a widely used affective EEG benchmark in which every participant experiences all conditions, we additionally used the DREAMER dataset [41]. DREAMER contains EEG recordings of 23 participants, each of whom watched the same 18 film clips and rated their valence and arousal on a scale from 1 to 5. The recordings were acquired with the Emotiv EPOC headset with the same 14 electrodes and the same sampling rate (128 Hz) as the EEG Violence Dataset, so KimiFormer was applied without any change to its input layer. For each clip, the last 60 s of the stimulus recording were divided into four non-overlapping 15-s windows (14 × 1920 samples, the same input size as in the main experiments), which gives 1656 windows. The valence and arousal ratings were binarized as high (>3) or low (≤3), and the two dimensions were treated as separate binary classification tasks. Following common practice for DREAMER, the signals were band-pass filtered between 1 and 45 Hz (fourth-order zero-phase Butterworth filter); the trial-specific baseline was removed by subtracting a 1-s template obtained by averaging the last 60 s of the resting baseline recorded before each clip; and each channel was z-score normalized with the mean and standard deviation of all stimulus data of the same participant (no labels are used in this step).

Evaluation followed a subject-independent LOSO protocol with 23 folds. In every fold, all windows of one participant formed the test set, and four of the remaining participants were held out as an inner validation set for early stopping; the test participant was therefore never used for training, early stopping or model selection. KimiFormer was compared with two representative raw-EEG deep models, EEGNet [30] and EEG-Conformer [34], which received the same input windows and were trained under the same protocol with a class-weighted cross-entropy loss (EEGNet with a learning rate of 1 × 10^−3^, as in its original implementation). The out-of-fold predictions of all folds were pooled, and accuracy, balanced accuracy (BAcc), macro-averaged F1-score and AUC were computed. Balanced accuracy was included because the binarized DREAMER labels are imbalanced (39.8% and 38.9% of the windows belong to the high-valence and high-arousal classes, respectively). Table 8 summarizes the comparison results. To account for between-subject variability, the balanced accuracy was also computed for each test participant, and 95% confidence intervals for the pooled balanced accuracy and AUC were obtained with a bootstrap over participants (2000 resamples), in which all windows of a resampled participant are kept together. The participant-level balanced accuracies were compared with chance (50%) and between the models with two-sided Wilcoxon signed-rank tests.

KimiFormer obtained the highest pooled value of every metric among the three raw-EEG models in both tasks. For valence, it reached an accuracy of 54.53% and a balanced accuracy of 52.71%, compared with 50.30% and 47.99% for EEG-Conformer and 49.09% and 49.12% for EEGNet. For arousal, KimiFormer reached an accuracy of 52.48% and a balanced accuracy of 50.20%, compared with 47.58% and 47.31% for EEG-Conformer and 49.09% and 48.19% for EEGNet. However, all balanced accuracies were close to the chance level of 50%, and the AUC of KimiFormer was 0.529 for valence and 0.499 for arousal. The 95% confidence intervals obtained with the bootstrap over participants included chance for all models and both tasks. At the participant level, the balanced accuracy of KimiFormer was 53.0 ± 9.2% (mean ± SD; range 37.5–71.9%) for valence and 50.9 ± 10.5% (20.8–75.0%) for arousal; it exceeded 50% for only 12 of the 23 participants in each task and did not differ significantly from chance (Wilcoxon signed-rank test, *p* = 0.27 and *p* = 0.57). The participant-level differences from EEGNet (+5.2 and +2.6 percentage points) and EEG-Conformer (+2.2 and +2.2 percentage points) were not significant either (*p* ≥ 0.14). The higher values of KimiFormer therefore represent a numerical difference from the comparison models and not evidence of effective subject-independent emotion recognition: under the LOSO protocol, none of the three models generalized reliably to unseen participants. This is consistent with the difficulty of subject-independent recognition on DREAMER: results obtained under LOSO protocols are not comparable with the much higher within-subject accuracies reported in the literature [37]. Moreover, KimiFormer was not tuned for DREAMER: no search for an optimal configuration was performed for this dataset, and the model was applied with the same hyperparameters as for the EEG Violence Dataset, because the purpose of this experiment was to compare it with established raw-EEG architectures under an identical protocol rather than to maximize its absolute performance on this dataset.

### 3.5. Analysis of Ocular, Muscle and Movement Artifacts

To account for the absence of an artifact rejection step in our pipeline, we examined whether ocular, muscle, or movement artifacts exhibited significant differences between the classes. For every segment, we computed ocular (0.5–4 Hz power and low-frequency peak-to-peak amplitude of the frontal channels AF3, AF4, F7 and F8), muscle (30–45 Hz power) and movement indices (maximum peak-to-peak amplitude and kurtosis) and compared the record means of the 38 neutral (control) and 47 violence records with Mann–Whitney U tests and Benjamini–Hochberg correction. The artifacts were not larger in the violence class but in the neutral (control) class (e.g., maximum peak-to-peak amplitude 908 vs. 482 µV, *p*(FDR) < 0.001), whereas the relative ocular and muscle power did not differ between the classes (*p*(FDR) ≥ 0.22); the differences therefore mainly reflect the overall signal amplitude, which is removed by the per-segment z-score normalization of the model input. When only the 4–30 Hz band power of the eight channels other than AF3, AF4, F7, F8, T7 and T8 was used, an RF classifier still reached an accuracy of 93.4% under the same LORO splits, and the LORO confidence of KimiFormer for each record was not correlated with any artifact index (|ρ| ≤ 0.13, *p* ≥ 0.24). Classifiers based only on the artifact indices also separated the classes (93.7%), indicating that the recordings of the two classes differ in several signal properties.

## 4. Discussion

The experimental results obtained on the EEG Violence Dataset demonstrate that the proposed KimiFormer architecture achieves high success in extracting discriminative features from raw EEG signals. This success is primarily due to the model’s STS module efficiently compressing the raw data. While traditional methods typically rely on hand-crafted features, the STS module has both optimized temporal resolution and automatically learned spatial correlations (SCs) between electrodes thanks to its hierarchical convolutional structure. The low variance across the folds of the 10-fold cross-validation indicates that this performance is stable across data partitions. The KimiFormer model employs attention mechanisms to capture rich contextual information and long-range dependencies. To improve computational efficiency at a moderate cost in accuracy, linear attention layers were incorporated in a 3:1 ratio, effectively reducing the overall computational requirements of the model.

### 4.1. Parameter Efficiency and Computing Power

The KimiFormer model not only classifies violence-induced affective states from wearable EEG signals, but also employs a lightweight architecture suited to on-device processing. The number of model parameters is lower than or comparable to that of many models in the literature whose suitability for real-time applications is debated. Moreover, thanks to its hybrid attention mechanism, the proposed model operates more efficiently in terms of computational power requirements by mitigating the *O*(*N*^2^) time complexity inherent to standard transformers.

The proposed model is compared with existing studies in the literature in terms of parameter count and computational power requirements. Table 9 presents a comparative analysis of the proposed KimiFormer model against several transformer and attention-inspired architectures in terms of parameter count and computational cost. As observed, conventional transformer models such as the Transformer Encoder (Base) and ViT-B/16 exhibit considerably high parameter counts and computational complexity. This makes them less suitable for resource-constrained environments. While more recent architectures have better optimizations, they still can be further improved. In contrast, the proposed KimiFormer model achieves remarkable efficiency with only 3.2 M parameters and 1.51 GFLOPs. Using a hybrid attention mechanism, KimiFormer maintains a balance between representation power and efficiency.

The results in Table 9 clearly show that the proposed KimiFormer model is more efficient than many existing transformer-based models. Traditional transformers and ViTs provide strong feature representation, but their large number of parameters and high computational cost make them difficult to use in real-time and resource-limited EEG applications. Although some recent models, such as Swin Transformer, Performer, and Conformer, aim to reduce this cost, they still require relatively high computational resources. In contrast, KimiFormer achieves a much lower parameter count and FLOPs while maintaining effective performance. This efficiency is mainly due to the hybrid attention mechanism, which reduces the computational cost of standard self-attention. As a result, KimiFormer is well suited to real-time affective state recognition from wearable EEG sensors: at 15.64 ms per 15-s segment on a Tesla T4 GPU, inference takes about 0.1% of the signal duration, leaving a large margin for continuous monitoring. Latency on edge hardware remains to be measured (Section 4.4), but the small parameter count (3.2 M) and computational cost (1.51 GFLOPs) make KimiFormer a practical candidate for lightweight, low-power systems.

### 4.2. Ablation Studies

To validate the effectiveness of the proposed KimiFormer model, we conducted an ablation study to examine the impact of different model components. Three case studies were designed to analyze the contributions of the hybrid attention mechanism, the STS module, and the convolutional module. To ensure a fair and direct comparison, all variations were trained for a fixed training duration (30 epochs) using the same optimization parameters (AdamW, lr = 1 × 10^−4^), and the same random seed for data split. During this process, to prevent model selection bias and compare the raw convergence rates of the architectures, the final performance and inference latency values at the end of the training period were used instead of selecting the best model on the validation set. These experiments were carried out on a single random split of the segments (70% training, 20% validation and 10% test; the split used for the hyperparameter exploration in Section 3.1), without the per-segment normalization, and were not nested within the record-wise folds of Section 3.3. They therefore indicate the relative contribution of the architectural components rather than the absolute performance of the model, which is given by the nested evaluation in Section 3.2 and Section 3.3.

Case 1: Effect of Hybrid Attention

In this case study, we conducted two different experiments (Case 1-1 and 1-2). To examine the effect of hybrid attention mechanisms on our model, we first used only linear attention layers and secondly used only full attention layers in KimiFormer. Then we compared the model sizes, required computational resources and accuracies.

Experimental results demonstrate that the hybrid attention mechanism provides a strong trade-off between accuracy and inference speed. When using only linear attention (KimiFormer-oLA), the model achieved a maximum accuracy of 97% with an inference latency of 15.59 ms. In contrast, a model using only full attention layers (KimiFormer-oFA) reached a maximum accuracy of 100% but with a higher latency of 17.97 ms. The hybrid attention mechanism also achieved 100% accuracy while reducing the latency to 15.64 ms. These results confirm that the hybrid approach improves accuracy compared to linear attention alone and reduces inference latency compared to full attention, achieving a more balanced overall performance. Because these results were obtained on a single random split of the segments, the attention variants were additionally evaluated under the record-wise LORO protocol of Section 3.3 with the training hyperparameters of Table 5 (Appendix A, Table A2). Under LORO, the full-attention-only model reached a higher accuracy (97.5%, 95% CI 95.7–99.0%) than the hybrid model (95.7%, 93.5–97.7%), whereas the linear-attention-only model reached 94.2% (91.3–96.6%); the 1:1 ratio, which lies between the evaluated 3:1 and full-attention configurations, was not re-evaluated under LORO. Under record-wise evaluation, the hybrid attention therefore does not match the accuracy of full attention; its advantage lies in the lower computational cost and inference latency, at the price of a moderate loss in accuracy, while it clearly improves on linear attention alone.

Case 2: Effect of the STS Module

In this case, we aimed to examine the effect of the STS module, which transforms raw EEG signals into high dimensional feature embeddings. The STS module is a vital part of the proposed KimiFormer model. To test its effectiveness, we evaluated the proposed STS module against two widely used baseline strategies: (Case 2-1) Raw Linear Projection (RLP), which applies a linear transformation to flattened channel data at each time step, and (Case 2-2) Linear Patch Embedding (LPE), the standard approach utilized in ViTs, which treats the signal as non-overlapping patches. As shown in Table 10, the two baseline approaches reached an accuracy of only about 65%, only slightly above the 60.7% majority-class baseline (442/728), indicating that these tokenizations learn almost nothing from raw EEG signals. In contrast, the model with the proposed STS module reached an accuracy of 100% on the same split. These results indicate that standard linear tokenization approaches are inadequate for modeling raw EEG data. By incorporating spatio-temporal structure, the STS module enables stable and effective representation learning.

Case 3: Effect of the Convolution Module

Although the attention mechanism is effective at modeling global dependencies, EEG signals contain short-term local patterns and high-frequency components that require localized feature extraction. To evaluate the role of the convolution module within the Conformer blocks, we compared the proposed KimiFormer model with a variant in which the convolution module was removed. This change effectively reduces the block to a standard Transformer encoder with Macaron-style FF modules. Although the Convolution Module is a standard component of the Conformer architecture [50], its specific contribution to EEG signal classification remains to be validated. Unlike speech signals (for which Conformer was originally designed), EEG signals have different spectral and temporal characteristics. Therefore, we performed an ablation study to investigate whether the inclusion of the Convolution Module provides a significant performance gain over a standard Transformer block. The results are summarized in Table 10.

Removing the convolution module (KimiFormer-oT) reduced the accuracy from 100% to 95.83% (Table 10), although it also reduced the number of parameters (2.77 M vs. 3.2 M) and the inference latency (11.89 vs. 15.64 ms). The reason for this result is that the convolution module introduces strong inductive biases for local feature extraction, which the pure transformer-based architecture lacks. By combining convolution with self-attention, KimiFormer benefits from local and long-range features. This combination allows the model to reach a higher accuracy than the transformer-only version.

The ablation studies show that the proposed KimiFormer model benefits from all components in the model. Table 10 summarizes the ablation study results obtained using a single random train/test split of the segments. As shown in Table 10, the STS module plays a very important role in converting raw EEG signals into integrated rich data. The results obtained after removing the STS module (Case 2-1, 2-2) show a significant decrease in accuracy. This demonstrates that the hierarchical implementation of STS’s STC and SC steps is highly effective in combining the temporal and spatial features of EEG signals. When attention mechanisms are compared on this split, although the ‘Full Attention Only’ (Case 1-2) configuration achieves an accuracy equivalent to the proposed model (100%), the proposed Hybrid structure reduces inference time by about 13% (from 17.97 ms to 15.64 ms per example). Furthermore, removing the convolution module within the main block (Case 3) increased speed by reducing the number of parameters to 2.77 M, but resulted in a 4.17-percentage-point loss in overall accuracy (95.83%). As a result, KimiFormer achieved the highest accuracy on this split, together with the full-attention-only variant, by combining the strong representation of the STS module, the computational efficiency of the Hybrid structure, and the local context capture capability of the Convolution module. Under the record-wise LORO evaluation, however, the full-attention-only variant was more accurate (see above), so that the hybrid structure should be regarded as a trade-off between accuracy and computational efficiency.

### 4.3. Comparison with Prior Work

To benchmark the performance of the proposed KimiFormer, we conducted a comparative analysis with recent studies. The baseline methods encompass a variety of deep learning paradigms, including CNNs, RNNs and hybrid architectures. Table 11 summarizes the classification accuracies and architectural characteristics reported in these studies alongside the results obtained by our proposed model.

Table 11 presents a comparison of the proposed KimiFormer with recent studies on stress and violence detection from EEG signals. Because these studies use different datasets and evaluation protocols, the values in Table 11 are not directly comparable, except for DMPat [45], which was evaluated on the same EEG Violence Dataset. DMPat extracts hand-crafted pattern features from the raw signal amplitudes and reported 100% accuracy with 10-fold and 98.49% with LORO cross-validation. When KimiFormer was trained under comparable conditions, i.e., on the raw signals without normalization and for a fixed number of 30 epochs without any selection on the test fold, it reached an accuracy of 99.3% (10-fold) and 96.4% (LORO) as an end-to-end model without any feature engineering. Under the stricter protocol used throughout this study, in which the electrode DC offset is removed by per-segment normalization and, under LORO, early stopping and the selection of the training hyperparameters are nested within the training records of each fold, KimiFormer reached 97.0% and 95.5%, respectively. Because the electrode DC level differs systematically between the records of the two classes, results obtained on raw amplitudes can partly reflect session- and participant-specific signal properties rather than neural activity; the normalized results therefore indicate what the model learns from the signal waveform itself. In addition, unlike complex ensemble methods (e.g., KRAFS-ANet) or generative models (e.g., AOS-GAN) that often require extensive computational resources or address specific imbalances, KimiFormer offers an end-to-end solution with a streamlined architecture. The balance between accuracy and computational efficiency makes the proposed model a suitable candidate for real-time EEG monitoring applications.

### 4.4. Limitations and Future Work

Despite the promising results achieved by KimiFormer, this study has limitations that should be taken into account. One limitation of this study is the dataset used in the experiments. Experiments were conducted on the EEG Violence Dataset, which involves a relatively limited number of subjects. While we employed different cross-validation techniques to control overfitting, further validation on larger-scale datasets is necessary to fully establish the model’s generalization capability. A second limitation is that, although the model achieves low inference latency, all experiments were performed on a GPU. Its deployment performance on edge devices, where environmental noise may pose additional challenges for EEG classification, remains to be examined in real-world scenarios. A further limitation concerns the structure of the EEG Violence Dataset. Each record contains EEG from only one stimulus condition, and the mapping between the 85 records and the 14 participants is not part of the public release. Consequently, a participant-wise evaluation cannot be constructed, and even under record-wise LORO cross-validation, participant- and session-specific signal properties are not balanced between the two classes; if, as the original description of the dataset suggests [13], the two conditions were recorded from different participants, the classifier may partly learn which group a recording belongs to rather than the affective state alone. Consistent with this, classifiers based only on artifact-related signal properties also separated the classes; although the artifacts were not larger in the violence class and the errors of KimiFormer were unrelated to the artifact level, a contribution of non-neural, recording-related signal components to the classification cannot be excluded completely. For the same reason, the segments of a record are not independent observations, and the effective sample size for generalization is the number of records, and ultimately the number of participants, rather than the number of segments. In addition, the class labels are derived from the stimulus condition; because the public release contains no self-reports or other manipulation checks, the induced affective states are assumed from the stimulus content and could not be verified for each participant. To complement the evaluation on this dataset, we evaluated KimiFormer on DREAMER, in which every participant watched all stimuli and rated every clip, so that the affective labels are not confounded with participant identity. Under a subject-independent LOSO protocol, however, KimiFormer and the two comparison models performed close to chance, and the participant-level confidence intervals included chance; the numerically higher values of KimiFormer therefore do not demonstrate subject-independent generalization. Consequently, the high record-wise accuracies on the EEG Violence Dataset should not be interpreted as evidence that the model recognizes affective states in unseen individuals. Methods for cross-subject generalization, such as data alignment, domain adaptation and domain generalization [36,37,38], and validation on larger datasets recorded with consumer-grade wearable headsets, in which every participant contributes to both conditions, will be needed to establish subject-independent generalization.

**Figure 15:**
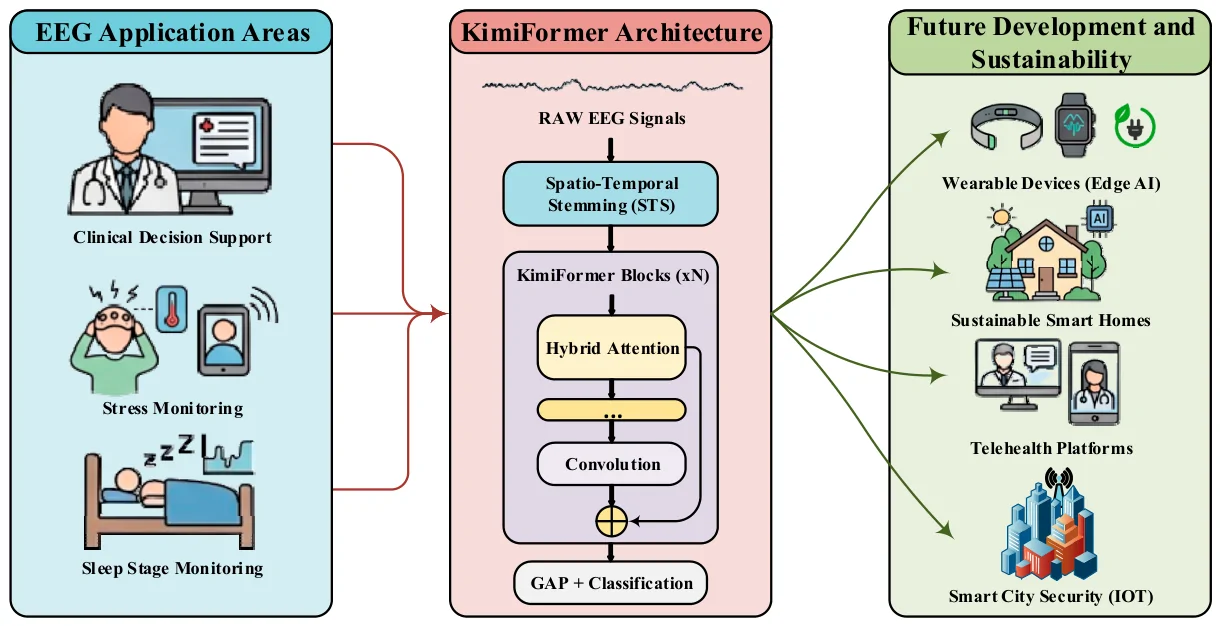
Potential EEG application areas and sustainable edge-deployment strategies for the KimiFormer architecture.

To address these limitations, future work will focus on two main topics. First, we aim to adapt the KimiFormer architecture for multi-class BCI tasks, such as sleep stage classification or emotion recognition, to verify its generalization across different EEG applications. Second, we plan to investigate model compression techniques, to develop a lightweight version of the KimiFormer model that is suitable for low-resource edge systems. This will contribute to the development of wearable, real-time stress and violence detection systems for public safety applications. Potential application areas and edge-deployment strategies for the KimiFormer architecture are illustrated in Figure 15.

## 5. Conclusions

In this study, we proposed KimiFormer, a novel Conformer-based hybrid-attention network. KimiFormer is designed for the classification of raw EEG signals. We used a hierarchical STS module to generate the rich context representations of EEG signals without any handcrafted preprocessing. One of the key advantages of the proposed model is its ability to effectively address the challenges of high dimensionality and low signal-to-noise ratio of raw EEG data. Another advantage of the proposed model is its ability to balance performance and computational requirements. The experimental results on the EEG Violence Dataset demonstrate the effectiveness of the proposed model.

The KimiFormer model achieves an accuracy of 97.0% under 10-fold and 95.5% under record-wise LORO cross-validation with nested hyperparameter selection. Ablation studies indicated the contribution of each component in our model. Record-wise evaluation reduces the risk of data leakage; however, with 14 participants from a single dataset and acquisition system, it does not demonstrate that the learned features generalize universally. Moreover, on the external DREAMER dataset, the subject-independent performance of KimiFormer, like that of EEGNet and EEG-Conformer, remained close to chance; subject-independent generalization has therefore not been demonstrated and requires further methodological work and validation on larger datasets. In our ablation studies, we showed that replacing the STS module with standard linear patching methods resulted in a drop in accuracy to ~65.3%. This indicates that the proposed STS module successfully processes the spatial and temporal features of the raw EEG data. Furthermore, the Hybrid Attention mechanism reduced the inference latency (from 17.97 ms to 15.64 ms) and the computational complexity compared with full attention; under the record-wise LORO evaluation, this efficiency came at the price of a moderate loss in accuracy compared with full attention (95.7% vs. 97.5%), whereas the hybrid model was clearly more accurate than linear attention alone (94.2%).

Under the same splits and the same input, KimiFormer achieved a higher accuracy, balanced accuracy and MCC than five feature-based classifiers, and on raw inputs it reached accuracies close to those of the feature-engineering pipeline DMPat on the same dataset without any feature engineering. These findings indicate that the proposed architecture is suitable for processing raw EEG data. The model represents an efficient end-to-end solution for real-time BCI systems.

In future studies, we aim to extend the KimiFormer architecture to multi-class BCI tasks, such as sleep stage classification and emotion recognition. Additionally, we plan to investigate lightweight quantization techniques to deploy the model on resource-constrained edge devices for wearable EEG monitoring.

## Figures and Tables

**Table 1:** Composition of the EEG Violence Dataset (records and 15-s segments per class).

Class	Record IDs	Records	Segments
Neutral (control)	1–23, 71–85	38	442
Violence	24–70	47	286
Total	1–85	85	728

**Table 2:** The transition table of the STS module.

Layer	Input	Operation	Output
STC	B, 1, 14, 1920	Conv2D: K = (3, 16), S = (1, 4), BN, GELU Activation, Padding = (1, 7)	B, 64, 14, 480
SC	B, 64, 14, 480	Conv2D: K = (14, 1), S = (1, 1), BN, GELU Activation	B, 128, 1, 480

**Table 3:** The transition table of the convolution module.

Layer	Input	Operation	Output
Pointwise Conv. (Expansion)	B, 128, 480	PwConv: K = 1, S = 1	B, 256, 480
GLU Activation	B, 256, 480	Splitting *A*, *B* = (128, 480) × 2 Multiplication = A ⊗ *σ*(*B*)	B, 128, 480
Depthwise Conv.	B, 128, 480	DwConv: K = 31, S = 1, BN, Swish Activation	B, 128, 480
Pointwise Conv. (Projection)	B, 128, 480	PwConv: K = 1, S = 1	B, 128, 480

**Table 4:** The transition table of the FF module.

Layer	Input	Operation	Output
Linear Layer	B, 480, 128	Dense, GELU, Dropout	B, 480, 512
Linear Layer	B, 480, 512	Dense	B, 480, 128

**Table 5:** Hyperparameters of the proposed model.

Category	Parameter	Value
Input Data	Input Shape (C × T)	14 × 1920
	Sampling Rate	128 Hz
STS Module	Stem Kernel Sizes	Layer 1: (3, 16), Layer 2: (14, 1)
	Stem Strides	Layer 1: (1, 4), Layer 2: (1, 1)
	Output Feature Size (T’)	480 (Time steps)
KimiFormer Encoder	Number of Blocks (N)	8
	Embedding Dimension (*d_model_*)	128
	Num. of Attention Heads (H)	8
	Attention Type Distribution	6 linear attention 2 full attention (Ratio 3:1)
	FFN Expansion Factor (E)	4
	Convolution Kernel Size (K)	31
	Activation Functions	GELU (FFN, Stem), Swish (ConvModule)
	Dropout Rate	0.1
Classification Head	Pooling strategy	Global Average Pooling
	Classifier Structure	Fully Connected
	Input-Output Sizes	Layer 1: (in: *d_model_*(128), out: *d_model/_2* (64)) Layer 2: (in: *d_model_/2*, out: num_classes)
	Number of Classes	2
Training Settings	Optimizer	AdamW
	Learning Rate	1 × 10^−4^
	Weight Decay	0.01
	Batch Size	32
	Number of Epochs	max. 50 (early stopping, patience = 10)
	Loss Function	Cross-Entropy Loss

**Table 6:** Hyperparameter search space and selected optimal values explored during model development on a single random split of the segments.

Hyperparameter	Evaluated Search Space	Selected Value
Learning Rate	{1 × 10^−3^, 1 × 10^−4^, 1 × 10^−5^}	1 × 10^−4^
Batch Size	{16, 32, 64}	32
Attention Ratio (Linear:Full)	{1:0, 3:1, 1:1, 0:1}	3:1
Convolutional Kernel Size	{15, 31, 63}	31
Number of KimiFormer Blocks (*N*)	{4, 6, 8, 10}	8

**Table 7:** Classification performance of KimiFormer and of feature-based baseline classifiers on the EEG Violence Dataset.

Model	Acc (%)	BAcc (%)	Prec (%)	Rec (%)	Spec (%)	F1 (%)	wF1 (%)	AUC	MCC
Segment-wise 10-fold cross-validation									
Logistic regression	94.2	94.4	90.7	95.1	93.7	92.8	94.3	0.979	0.881
k-nearest neighbors	94.6	95.2	89.7	**97.6**	92.8	93.5	94.7	0.990	0.892
Linear discriminant analysis	93.8	94.1	89.5	95.5	92.8	92.4	93.9	0.978	0.873
SVM (RBF kernel)	96.0	95.7	95.7	94.1	97.3	94.9	96.0	0.991	0.916
Random forest	96.7	96.3	**97.1**	94.4	**98.2**	95.7	96.7	**0.993**	0.931
KimiFormer (raw EEG, end-to-end)	**97.0**	**96.8**	96.5	95.8	97.7	**96.1**	**97.0**	0.992	**0.937**
Record-wise LORO cross-validation									
Logistic regression	90.1	90.8	83.0	94.1	87.6	88.2	90.2	0.934	0.802
k-nearest neighbors	87.9	89.4	78.0	**96.5**	82.4	86.2	88.1	0.964	0.771
Linear discriminant analysis	87.9	89.0	79.1	94.1	83.9	85.9	88.0	0.924	0.763
SVM (RBF kernel)	93.1	93.0	90.1	92.7	93.4	91.4	93.1	0.978	0.857
Random forest	94.8	94.5	93.7	93.0	95.9	93.3	94.8	**0.985**	0.890
KimiFormer (raw EEG, end-to-end)	**95.5**	**95.3**	**93.8**	94.8	**95.9**	**94.3**	**95.5**	0.981	**0.905**

Acc, accuracy; BAcc, balanced accuracy; Prec, precision; Rec, recall (sensitivity); Spec, specificity; F1, F1-score of the violence class; wF1, weighted F1-score; AUC, area under the ROC curve; MCC, Matthews correlation coefficient. Record-wise LORO results of KimiFormer were obtained with nested selection of the learning rate and batch size. The best value of each metric within each validation scheme is shown in bold.

**Table 8:** Subject-independent (leave-one-subject-out, 23 folds) comparison of raw-EEG deep models on the DREAMER dataset (1656 windows of 15 s; pooled out-of-fold predictions; 95% confidence intervals from a bootstrap over participants in parentheses).

Task	Model	Acc (%)	BAcc (%)	Macro-F1 (%)	AUC
Valence	EEGNet [30]	49.09	49.12 (46.3–51.8)	48.55	0.488 (0.449–0.523)
	EEG-Conformer [34]	50.30	47.99 (43.8–52.6)	47.99	0.478 (0.430–0.529)
	KimiFormer (ours)	54.53	52.71 (48.9–56.8)	52.68	0.529 (0.482–0.577)
Arousal	EEGNet [30]	49.09	48.19 (45.9–50.4)	48.19	0.471 (0.434–0.506)
	EEG-Conformer [34]	47.58	47.31 (42.0–52.6)	47.24	0.484 (0.420–0.551)
	KimiFormer (ours)	52.48	50.20 (45.0–55.0)	49.45	0.499 (0.437–0.561)

Acc, accuracy; BAcc, balanced accuracy; AUC, area under the ROC curve. Participant-level balanced accuracy (mean ± SD over the 23 participants): valence 47.8 ± 8.3%, 50.8 ± 10.3% and 53.0 ± 9.2%, arousal 48.3 ± 6.3%, 48.7 ± 7.0% and 50.9 ± 10.5% for EEGNet, EEG-Conformer and KimiFormer, respectively; per-participant results are given in Table A3.

**Table 9:** Comparison of the proposed model with transformer and attention-inspired models in terms of parameter count and computational cost.

Model	Architecture	Params (M)	FLOPs (GFLOPs)
Transformer Encoder (Base)	Self-Attention	~65.0	~4.5
ViT-B/16	Vision Transformer	~86.0	~17.6
Swin Transformer (Tiny)	Hierarchical Att.	~28.0	~4.5
Conformer (Base)	CNN + Attention	~10–12	~2.2
Performer	Linear Attention	~20.0	~3.8
ConvNeXt-Tiny	Convolution	~28.6	~4.5
KimiFormer (Proposed)	Hybrid Attention	3.2	1.51

**Table 10:** Comparison table of ablation studies.

Case	Configuration	Modification	Accuracy (%)	Parameters (M)	Speed (ms/Sample)
1-1	KimiFormer-oLA	Only Linear Attentions	97	3.2	15.59
1-2	KimiFormer-oFA	Only Full Attentions	**100**	3.2	17.97
2-1	KimiFormer-RLP	Raw Linear Projection	65.28	3.09	14.38
2-2	KimiFormer-LPE	Linear Patch Embedding	65.30	3.09	15.40
3	KimiFormer-oT	Only Transformer main blocks	95.83	**2.77**	**11.89**
Proposed	KimiFormer	The proposed configuration	**100**	3.2	15.64

The best value in each column (highest accuracy, fewest parameters and lowest inference time) is shown in bold.

**Table 11:** Comparison of the proposed model with the literature.

Model	Method	Aim of the Study	Dataset	Results
CubicPat [14]	Cubic Pattern (CubicPat) + CWINCA + tkNN	Detecting mental performance and stress from EEG signals	Mental Performance Dataset (3949 recordings)	99.70% Accuracy,
			Earthquake Stress Dataset (3667 recordings) 2 classes	96.26% Accuracy
AOS-GAN [24]	FA-FCN (Segmentation) + HW-STFT (Feature Ext.) + RLASSO + AOS-GAN	Detecting stress in imbalanced conditions	PhysioNet mental Arithmetic Task with 2 classes	94% Accuracy, 91% Precision, 92% Recall, 96% F1 Score
KRAFS-ANet [16]	NMI + RFE (Channel selection) + Bagging (KNN, RF, SVM) + ANN Meta-Classifier	Stress classification with optimum channel selection	MAT (2 classes)	98.63% Accuracy
			SAM40 (2 classes)	97.25% Accuracy
			DASPS (2 classes)	94.92% Accuracy
StressNet [19]	Azimuthal Projection + 2D CNN + LSTM	Detecting stress states by converting EEG signals into azimuthal projection images using a hybrid DL model	DEAP and SEED datasets; 380 total samples; 2 classes	97.8% Accuracy 94.0% Specificity 94.2% Sensitivity
VGGish-CNN [17]	VGGish (Feature Extraction) + CNN (Classification)	Stress detection using convolution neural network	SAM 40 dataset; 40 subjects (480 signals segmented into images); 4 classes	99.25% Accuracy 99.75% Specificity
DMPat [45]	Distance Matrix Pattern (DMPat) + INCA + tkNN	Violence detection from EEG signals	EEG Violence Dataset; 14 participants (85 records); 2 classes	100% Accuracy (10-fold), 98.49% Accuracy (LORO CV)
Hybrid DL Model [21]	CNN (Spatial features) + MLP (Classification)	Multiclass stress detection from EEG signals	EEG Brainwave Dataset (Kaggle); 2380 samples; 3 classes (Normal, Mild, High)	99.40% Val. Accuracy, 99.00% F1-Score
KimiFormer	Conformer-based Hybrid Attention	Violence induced affective state recognition from raw EEG signals	EEG Violence Dataset; 14 participants (85 records); 728 non-overlapping 15-s segments (442 neutral (control), 286 violence), 2 classes	97.0% Accuracy (10-fold), 95.5% Accuracy (nested LORO CV), 95.5% Weighted F1 (LORO CV); raw input, no normalization: 99.3% (10-fold)/96.4% (LORO)

## Data Availability

The EEG Violence Dataset analyzed in this study is available at https://www.kaggle.com/datasets/turkertuncer/turkish-violence-detection-eeg-dataset (accessed on 20 October 2026). The DREAMER dataset [41] is available from its authors upon request and was used in accordance with its terms of use.

## Abbreviations

The following abbreviations are used in this manuscript:

ANN	Artificial Neural Network
AUC	Area Under the ROC Curve
CI	Confidence Interval
BCI	Brain–Computer Interface
BN	Batch Normalization
CNN	Convolutional Neural Network
CV	Cross-Validation
EEG	Electroencephalography
FF/FFN	Feed-Forward/Feed-Forward Network
FDR	False Discovery Rate
FLA	Flash Linear Attention
FPS	Frames Per Second
GAP	Global Average Pooling
GELU	Gaussian Error Linear Unit
GFLOPs	Giga Floating-Point Operations
GLU	Gated Linear Unit
GPU	Graphic Processing Unit
GRU	Gated Recurrent Unit
IoT	Internet of Things
KNN	K-Nearest Neighbors
LN	Layer Normalization
LORO	Leave-One-Record-Out
LOSO	Leave-One-Subject-Out
LPE	Linear Patch Embedding
LSTM	Long Short-Term Memory
MCC	Matthews Correlation Coefficient
MLP	Multilayer Perceptron
NMI	Normalized Mutual Information
RBF	Radial Basis Function
RF	Random Forest
RFE	Recursive Feature Elimination
RLP	Raw Linear Projection
RNN	Recurrent Neural Network
ROC	Receiver Operating Characteristic
SC	Spatial Collapse
STC	Spatio-Temporal Convolution
STS	Spatio-Temporal Stemming
SVM	Support Vector Machine
ViT	Vision Transformer

## Appendix A

**Table A1:** Records of the EEG Violence Dataset by class and number of 15-s segments per record.

Class	Segments per Record	Records	Record IDs
Neutral (control)	12	34	1–20, 22, 72–84
	11	2	23, 85
	7	1	21
	5	1	71
Violence	8	10	30, 33–36, 43, 53, 56, 57, 66
	7	4	32, 44, 55, 67
	6	13	25, 27–29, 31, 41, 42, 48, 50–52, 64, 65
	5	20	24, 26, 37–40, 45–47, 49, 54, 58–63, 68–70
Total	5–12	85	1–85 (728 segments)

**Table A2:** Record-wise LORO results (85 folds, pooled out-of-fold predictions) of KimiFormer for each candidate of the nested hyperparameter selection when it is used unchanged in all folds (sensitivity analysis), and of the nested selection; lower part: architectural and attention variants, each used unchanged in all folds; 95% CIs: cluster bootstrap over records (2000 resamples).

Learning Rate	Batch Size	Selected in Folds	Acc (%) [95% CI]	BAcc (%) [95% CI]	MCC [95% CI]	AUC
1 × 10^−3^	16	18	94.1 [90.9–96.6]	93.7 [90.5–96.4]	0.876 [0.812–0.930]	0.976
1 × 10^−3^	32	17	96.3 [94.0–98.1]	96.0 [93.5–98.1]	0.922 [0.874–0.960]	0.982
1 × 10^−3^	64	15	94.6 [91.7–97.1]	94.4 [91.5–96.8]	0.888 [0.825–0.940]	0.978
1 × 10^−4^	16	18	95.6 [93.3–97.6]	95.6 [93.2–97.6]	0.908 [0.859–0.949]	0.982
1 × 10^−4^	32 *	10	95.7 [93.5–97.7]	95.6 [93.2–97.7]	0.911 [0.861–0.951]	0.982
1 × 10^−4^	64	3	97.0 [95.2–98.4]	96.9 [95.2–98.4]	0.937 [0.900–0.967]	0.988
1 × 10^−5^	16	4	95.5 [93.2–97.5]	95.3 [93.0–97.4]	0.905 [0.856–0.948]	0.982
1 × 10^−5^	32	0	95.2 [92.9–97.2]	94.9 [92.4–97.2]	0.899 [0.851–0.941]	0.982
1 × 10^−5^	64	0	95.3 [93.1–97.3]	95.1 [92.7–97.2]	0.902 [0.853–0.943]	0.983
**Nested selection**		**85**	**95.5 [92.7–97.6]**	**95.3 [92.4–97.6]**	**0.905 [0.847–0.951]**	**0.981**
Architectural and attention variants (learning rate 1 × 10^−4^, batch size 32), each used unchanged in all folds						
N = 4		–	96.8 [95.2–98.4]	96.5 [94.6–98.3]	0.934 [0.898–0.965]	0.984
N = 6		–	96.3 [94.5–97.9]	95.8 [94.0–97.5]	0.922 [0.886–0.954]	0.979
N = 10		–	95.1 [92.7–97.1]	94.8 [92.3–96.8]	0.896 [0.847–0.937]	0.966
Kernel size 15		–	96.0 [94.1–97.7]	95.7 [93.6–97.6]	0.916 [0.877–0.952]	0.984
Kernel size 63		–	95.3 [92.9–97.3]	95.1 [92.7–97.1]	0.902 [0.852–0.944]	0.981
Linear attention only		–	94.2 [91.3–96.6]	93.8 [90.7–96.4]	0.879 [0.816–0.929]	0.976
Full attention only		–	97.5 [95.7–99.0]	97.6 [95.6–99.1]	0.949 [0.910–0.979]	0.986

* Configuration of Table 5 (used in all folds in the first version of this study). Acc, accuracy; BAcc, balanced accuracy; MCC, Matthews correlation coefficient; AUC, area under the ROC curve. The candidate results are a sensitivity analysis; choosing the best candidate from this table would use the test records for model selection. The nested selection row (in bold) is the performance estimate reported in the main text.

**Table A3:** Balanced accuracy (%) of each test participant (ID) in the subject-independent LOSO evaluation on the DREAMER dataset (72 windows per participant). High (%), percentage of the participant’s windows labeled high valence or high arousal.

ID	Valence				Arousal			
	High (%)	EEGNet	EEG-Conformer	KimiFormer	High (%)	EEGNet	EEG-Conformer	KimiFormer
1	50.0	51.4	50.0	48.6	27.8	38.8	53.8	56.2
2	27.8	46.7	50.0	63.7	50.0	56.9	47.2	52.8
3	44.4	52.2	50.0	51.2	38.9	53.2	50.0	51.0
4	22.2	19.6	17.9	37.5	27.8	37.1	31.3	75.0
5	33.3	52.1	50.0	45.8	44.4	57.2	62.5	53.8
6	44.4	53.4	50.0	48.1	66.7	55.2	47.9	40.6
7	27.8	42.1	73.7	71.2	38.9	55.5	62.7	63.3
8	33.3	52.1	63.5	55.2	27.8	51.3	45.2	45.0
9	44.4	49.4	51.6	58.8	72.2	46.2	53.7	49.8
10	66.7	35.4	50.0	52.1	38.9	51.3	50.6	45.5
11	33.3	43.8	51.0	66.7	38.9	45.1	50.0	52.4
12	50.0	47.2	41.7	51.4	44.4	39.7	48.8	50.0
13	55.6	54.1	48.8	43.4	44.4	47.8	37.2	65.9
14	44.4	45.6	50.0	50.0	33.3	51.0	50.0	40.6
15	27.8	52.3	57.5	42.1	50.0	44.4	47.2	50.0
16	33.3	43.8	56.2	65.6	44.4	47.8	48.4	47.5
17	33.3	51.0	50.0	47.9	27.8	42.5	50.0	51.2
18	33.3	49.0	66.7	71.9	50.0	51.4	48.6	51.4
19	50.0	65.3	52.8	52.8	77.8	55.8	36.2	49.6
20	33.3	44.8	53.1	52.1	33.3	44.8	50.0	54.2
21	33.3	51.0	44.8	49.0	61.1	37.5	47.4	62.7
22	38.9	46.8	48.1	48.4	33.3	50.0	52.1	20.8
23	44.4	50.3	41.2	45.3	33.3	51.0	50.0	42.7
Mean	39.4	47.8	50.8	53.0	43.7	48.3	48.7	50.9
SD		8.3	10.3	9.2		6.3	7.0	10.5

Mean and SD (standard deviation) over the 23 participants. A balanced accuracy of 50% corresponds to chance.
